# Ultrasound-driven programmable artificial muscles

**DOI:** 10.1038/s41586-025-09650-3

**Published:** 2025-10-29

**Authors:** Zhan Shi, Zhiyuan Zhang, Justus Schnermann, Stephan C. F. Neuhauss, Nitesh Nama, Raphael Wittkowski, Daniel Ahmed

**Affiliations:** 1 Acoustic Robotics Systems Laboratory, Institute of Robotics and Intelligent Systems, Department of Mechanical and Process Engineering, ETH Zürich, Zurich, Switzerland; 2 Institute of Theoretical Physics, Center for Soft Nanoscience, University of Münster, Münster, Germany; 3 Department of Molecular Life Sciences, University of Zürich, Zurich, Switzerland; 4 Department of Mechanical and Materials Engineering, University of Nebraska-Lincoln, Lincoln, NE USA; 5 Department of Physics, RWTH Aachen University, Aachen, Germany; 6 DWI - Leibniz Institute for Interactive Materials, Aachen, Germany

**Keywords:** Mechanical engineering, Acoustics

## Abstract

Muscular systems^1^, the fundamental components of mobility in animals, have sparked innovations across technological and medical fields^2,3^. Yet artificial muscles suffer from dynamic programmability, scalability and responsiveness owing to complex actuation mechanisms and demanding material requirements. Here we introduce a design paradigm for artificial muscles, utilizing more than 10,000 microbubbles with targeted ultrasound activation. These microbubbles are engineered with precise dimensions that correspond to distinct resonance frequencies. When stimulated by a sweeping-frequency ultrasound, microbubble arrays in the artificial muscle undergo selective oscillations and generate distributed point thrusts, enabling the muscle to achieve programmable deformation with remarkable attributes: a high compactness of approximately 3,000 microbubbles per mm^2^, a low weight of 0.047 mg mm^−2^, a substantial force intensity of approximately 7.6 μN mm^−2^ and fast response (sub-100 ms during gripping). Moreover, they offer good scalability (from micrometre to centimetre scale), exceptional compliance and many degrees of freedom. We support our approach with a theoretical model and demonstrate applications spanning flexible organism manipulation, conformable robotic skins for adding mobility to static objects and conformally attaching to ex vivo porcine organs, and biomimetic stingraybots for propulsion within ex vivo biological environments. The customizable artificial muscles could offer both immediate and long-term impact on soft robotics, wearable technologies, haptics and biomedical instrumentation.

## Abstract (web summary)

A soft artificial muscle with programmable actuation is achieved using microbubble arrays and targeted ultrasound activation, with potential applications in soft robotics, wearable technologies, haptics and biomedical instrumentation.

## Main

Flexible, compact and adaptive artificial muscles are set to be transformative across multiple fields, including soft robotics^4,5^, wearables for human–machine interactions and healthcare, such as prosthetics^6^, orthotics^7^ and embodied sensing^8,9^, and assistance in sophisticated manufacturing through dexterous manipulation^10,11^. In biomedicine, they could revolutionize soft surgical tools^12^, implantable electrodes^13^ and artificial organs such as the heart^14^. Despite their potential, current artificial muscles such as tendon-based^15^ and pneumatic types^16^ encounter substantial challenges in wireless control, integration and miniaturization owing to dependencies on tethering, complex operational mechanisms and large input requirements. Although external stimuli such as chemicals^17^, light^18,19^, temperature^20–22^, electric fields^23–25^ and magnetic fields^26,27^ have been deployed for wireless actuation, they face challenges in biocompatibility, spatial resolution and dynamic programmability. Chemical methods often require fuels that could be toxic^28^, light-based systems suffer from limited tissue penetration and potential thermal damage^29^, and magnetic systems necessitate bulky hardware while risking Joule heating^30^. By contrast, acoustic actuation emerges as a promising biocompatible alternative. It offers a material-independent and simplified design, enabling wireless control, remote deployment, millisecond-scale responsiveness, multimodal programmability, high spatial selectivity and deep tissue penetration—all without invasive hardware. Moreover, its compatibility with existing clinical ultrasound devices and imaging systems makes it particularly uniquely suited for in vivo use and broader biomedical applications^31–37^.

Central to this approach are resonant microbubbles, which concentrate acoustic energy and enable weak ultrasound sources to generate amplified responses. Although previous ultrasound-actuated microrobots and actuators have used single or sparse microbubbles embedded in polymers to achieve basic propulsion^38–40^, their functionality remained limited. Directional steering has been demonstrated through strategies such as tuning microbubble sizes^41^, applying magnetic navigation^42^ or hybrid methods that combine magnetic fields with asymmetric appendages in encapsulated shells^43^. An actuator composed of a microbubble attached to a flexible beam was developed to analyse the kinematic behaviour of simple microstructures through the excitation of different pairs of bubble actuator modules^44^. Another study used arrays of microbubbles integrated onto centimetre-scale rigid substrates to induce bi-rotational motion^45^, demonstrating potential applications in endoscope design^46^. However, these systems lack the programmability, scalability and dynamic adaptability required to emulate natural muscle behaviour. Critically, to the best of our knowledge, no previous work has achieved ultrasound-actuated soft artificial muscles, marking a significant gap in biologically inspired actuation technologies.

A reason why ultrasound-based artificial muscles have remained undeveloped is that soft materials typically have low acoustic contrast factors compared with water, leading to inadequate force generation for efficient functionality when activated by ultrasound. This predicament is exacerbated by a lack of understanding of the interactions between sound and complex soft materials, impeding the progress of effective sound-driven muscle systems. However, we found that integrating ultrasound-activated microbubble arrays into soft artificial muscles presents a clever approach that could potentially address these limitations.

Here we introduce an artificial muscle built on acoustically activated microbubble arrays. This synthetic muscle comprises a thin, transparent and flexible membrane that houses over 10,000 microcavities arranged in arrays, designed to confine microbubbles of various sizes. When these microbubbles are acoustically stimulated, they generate thrust, causing the membrane to deform. Tailored activation of differently sized microbubble arrays through programmable sweeping-frequency ultrasound excitation results in localized point forces, allowing dynamic multimodal deformation of the artificial muscle. The tunable nature and scalability of these microbubble arrays herald an era of possibilities, positioning these acoustic artificial muscles at the forefront of innovation in robotics, wearable technology, prosthetic development and soft surgical devices.

## Design and fabrication

**Fig. 1:**
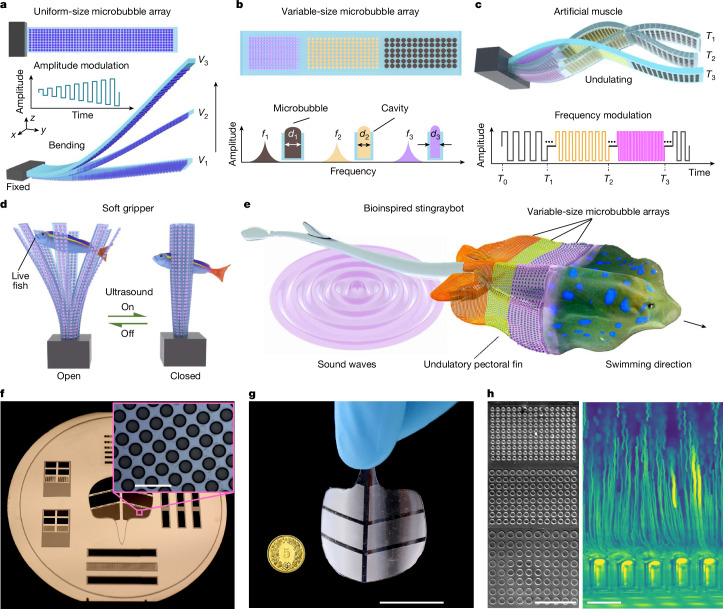
Ultrasound-actuated microbubble-array artificial muscles. **a**, A uniform-size microbubble-array artificial muscle consists of thousands of microbubbles on its bottom surface. Under continuous ultrasound excitation, the artificial muscle bends upwards with different excitation voltages, labelled as *V*_1_, *V*_2_ and *V*_3_. Inset: the input ultrasound signal with modulated amplitude versus time. **b**, A variable-size microbubble-array artificial muscle comprises three microbubble arrays with different diameters (*d*), each corresponding to a distinct natural frequency (*f*) and represented by the colours purple, yellow and grey. **c**, Under sweeping-frequency ultrasound excitation, the artificial muscle exhibits multimodal deformation in the time domain, shown at time points *T*_1_, *T*_2_ and *T*_3_. **d**, Schematic of a soft gripper constructed with an array of artificial muscles patterned with uniform-size microbubble arrays. Upon ultrasound excitation, these muscles close simultaneously in milliseconds. **e**, Schematic of a bioinspired stingraybot incorporating variable-size microbubble-array artificial muscles. Under sweeping-frequency ultrasound excitation, the stingraybot enacts undulating propulsion. **f**, A silicon wafer with micropillar arrays serves as the negative mould of microbubble cavities in standard soft-lithography fabrication. Inset: the micropillar array. **g**, A prototype of the stingraybot near a 5-cent Swiss franc coin. **h**, Left: trapped microbubble arrays. Right: upwards microstreaming jets generated from a microbubble array oscillating under ultrasound excitation visualized by 6-μm-diameter tracer microparticles. *n* = 3 independent samples. Scale bars, 300 μm (**f**, inset), 2 cm (**g**), 500 μm (**h**, left), 100 μm (**h**, right).

In the initial design of the ultrasound-driven artificial muscle (Fig. 1a), we incorporated uniform-size microcavities on the muscle’s bottom surface. When the muscle was submerged in an acoustic chamber filled with water, it resulted in the simultaneous trapping of tens of thousands of gas-filled microbubbles within these cavities, a phenomenon driven by surface tension. To test the muscle’s actuation, we anchored one end of the muscle and left the other free, forming a cantilever configuration. Subsequently, we activated a piezoelectric transducer to generate ultrasound. The incident sound waves propagated through the liquid, triggering oscillations in the microbubbles. As all microbubbles in the muscle were of identical dimensions, they were simultaneously excited. This harmonic bubble oscillation generated collective acoustic streaming and radiation forces, applying a uniform opposing force to the muscle’s bottom surface and resulting in its upwards flexion. By modulating the ultrasound excitation voltage, we controlled the deformation amplitude of the artificial muscle.

We then designed an artificial muscle featuring microbubble arrays of varying bubble sizes, illustrated in Fig. 1b. As microbubbles of different sizes show distinct resonance frequencies, they can be independently activated to produce localized opposing forces and selective muscle deformation. By applying a sweeping-frequency ultrasound signal that encompasses the natural frequencies of all microbubbles, we sequentially activated distinct arrays along the muscle’s longitudinal axis. This orchestrated activation generated complex undulatory motion across multiple excitation cycles (Fig. 1c). Thus, in implementing this arrangement of microbubbles of various sizes and their frequency-selective excitation through ultrasound modulation, we have unlocked a capability to control multimodal deformations. The versatility of these artificial muscles facilitates a wide array of applications. For example, we implemented these artificial muscles in the development of a soft gripper, crafted to delicately handle live fish (Fig. 1d), and in the design of soft swimmers as surgical soft robots, inspired by the form and function of stingrays (Fig. 1e), among other functional systems.

Prototypes of these artificial muscles were fabricated using a high-resolution mould replica method. First, micropillar arrays were patterned on a silicon wafer using soft lithography to serve as negative moulds for cylindrical microcavities (Fig. 1f). All pillars were designed with identical heights and spacings, corresponding to the dimensions of the desired microbubbles (Supplementary Fig. 1). A thin layer of polydimethylsiloxane (PDMS) was then spin-coated onto the wafer, yielding thin membranes with uniform thicknesses ranging from 80 μm to 250 μm (Supplementary Fig. 2). After curing, these artificial muscles including the artificial stingray (Fig. 1g) were demoulded, sectioned and prepared for testing. Full fabrication details are provided in Methods. Figure 1h shows trapped microbubble arrays and the upwards microstreaming jets produced during ultrasound excitation.

## Characterization of microbubble arrays

To advance our understanding and control of microbubble arrays in artificial muscles, we observed the transient dynamics of microbubbles using a high-speed camera while applying acoustic fields with excitation frequencies ranging from 1 kHz to 100 kHz and peak-to-peak (PP) voltage amplitudes of 10 V_PP_ to 60 V_PP_ in square waveforms. Further details of the acoustic set-up are provided in Methods.

**Extended Data Fig. 1:**
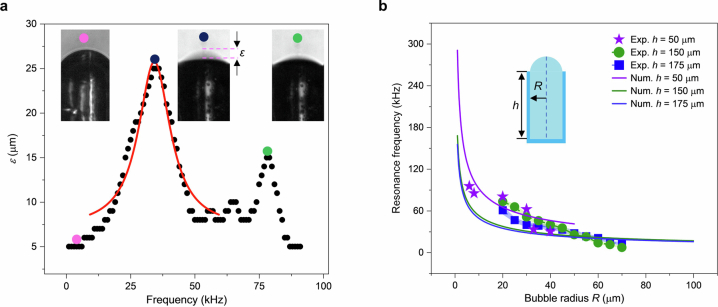
Characterization of the resonance frequencies of differently sized microbubbles. **a**, Oscillation amplitude of a 60 μm × 150 μm microbubble as a function of the ultrasound excitation frequency. According to the measured oscillation amplitude of the microbubbles, the resonance frequency is identified to be 35.5 kHz. **b**, Experimentally measured resonance frequencies of microbubbles with depths of 150 μm and 175 μm, radii ranging from 20 to 70 μm in 5 μm increments, and a depth of 50 μm with radii of 6 μm, 8 μm, 20 μm, 30 μm, 33 μm and 40 μm. The solid lines represent numerically predicted results. Additionally, notable discrepancies exist between the calculated and measured resonance frequencies, which may originate from transducer coupling with the glass slide, interactions between bubbles, or other factors.

**Extended Data Fig. 2:**
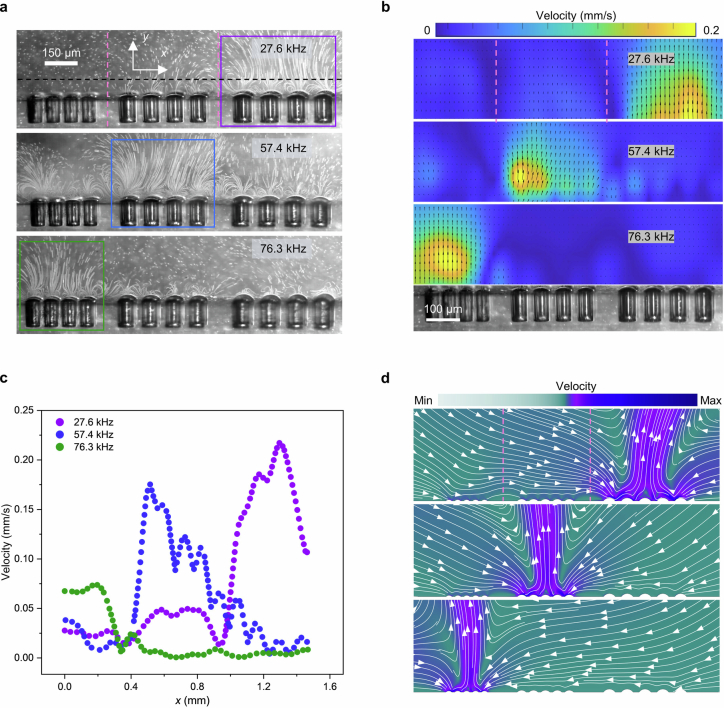
Selective actuation of microbubble arrays. **a**, Microstreaming respectively generated by 40 μm, 60 μm, and 80 μm microbubble arrays under ultrasound frequencies of 76.3 kHz (green), 57.4 kHz (blue), and 27.6 kHz (purple). Colored boxes indicate the activated microbubbles. The black dashed line denotes the measurement position of the microstreaming velocity, which is 100 μm away from the surface. **b**, Measured streaming flow of **a** with 2 μm tracer microparticles. The streaming flow field was analyzed by PIV (Matlab R2022b, PIVlab 2.60). The color bar denotes the particle moving velocity perpendicular to and extending away from the bubble surface. **c**, Plot of the measured microstreaming velocity along the long axis of the variable-size microbubble array under different excitation frequencies. **d**, Simulations of microstreaming around the bubble array with three different excitation frequencies. The pink dotted lines in **a**, **b**, and **d** delineate the boundaries of three distinct microbubble arrays.

We began by identifying the resonance frequencies of microbubbles confined within cavities of different diameters (40−140 μm, in 10-μm increments) and depths (50 μm, 150 μm and 175 μm) while maintaining a constant excitation voltage of 15 V_PP_. Resonance frequencies were identified by locating peak oscillation amplitudes during frequency sweeps (Extended Data Fig. 1a). As shown in Extended Data Fig. 1b, resonance frequencies decreased from 95.5 kHz to 8.9 kHz with increasing microbubble diameters, consistent with the inverse scaling relationship between natural frequency and the bubble diameter^47^. In addition, microbubbles with depths of 50 μm, 150 μm and 175 μm showed a decrease in resonance frequencies, indicating that the bubble depth also affects oscillation. We further investigated the selective actuation of variable-size microbubble arrays with cavities of 40 μm, 60 μm and 80 μm diameter, each 150 μm in depth, integrated within a single miniaturized artificial muscle (500 μm × 500 μm × 200 μm) with corresponding frequencies (76.3 kHz, 57.4 kHz and 27.6 kHz, respectively), as shown in Extended Data Fig. 2 and Supplementary Video 1. The distinct resonance profiles of microbubbles across sizes enable selective ultrasound excitation, forming the basis for programmable microbubble arrays. Detailed microstreaming characterization is provided in Methods.

## Programmable actuation

The versatility of microbubble arrays in terms of programmability and selectivity enables an innovative approach for designing soft actuators with enhanced flexibility and control. To verify that microbubble oscillation is the dominant driver of this muscle bending, we systematically varied the transducer’s position relative to the microbubble-embedded side of the artificial muscle (3 cm × 0.5 cm × 80 μm), which contains over 10,000 uniform microbubbles within cavities (40 μm diameter, 50 μm depth). The transducer was positioned with four distinct orientations: (1) directly facing the microbubble-embedded side, (2) opposite to it, and (3) and (4) perpendicular to the array’s left and right sides of the artificial muscle (Supplementary Fig. 3 and Supplementary Video 2). When activated at 80.5 kHz and 60 V_PP_, the muscle consistently bent away in the direction opposite to the microbubble-array side, across all configurations, despite variations in bending amplitudes. This directional uniformity confirms that microbubble-generated reverse thrust is the primary force driving the deformation. More control experiments and characterization of artificial muscle deformation are provided in Methods.

**Fig. 2:**
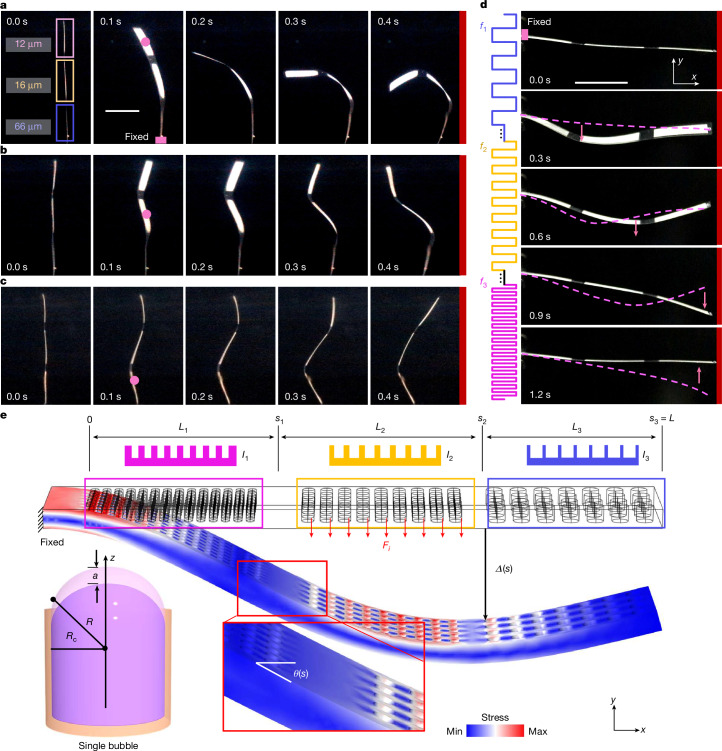
Actuation and modelling of microbubble-array artificial muscles. **a**–**c**, Time-lapse images of the selective deformation shapes of a variable-size microbubble-array artificial muscle (3 cm × 0.5 cm × 80 μm) containing microbubbles of diameter 12 μm, 16 μm and 66 μm, each 50 μm in depth, excited at 96.5 kHz (**a**), 82.3 kHz (**b**) and 33.2 kHz (**c**), respectively, at 60 V_PP_. The pink dots indicate the region of the bubble array being activated. **d**, Time-lapse images of the variable-size microbubble-array artificial muscle under sweeping-frequency ultrasound excitation (20–90 kHz, 1.2 s, 60 V_PP_). The pink dashed lines mark the shape of the muscle at the previous time step and the pink arrows mark the bending direction of the excited part. **e**, Modelling of the activation mechanism of microbubble-array artificial muscles. The pink, yellow and blue boxes represent differently sized microbubble-array segments. The upper portion illustrates schematics of the cross-section of the artificial muscle, each part of the artificial muscle corresponding to a specific length (*L*) and second moment of area (*I*). *F*_*i*_ denotes the thrust force generated by the microstreaming (here the yellow segment of the muscle generates thrust), *Δ* and *θ* denote the deflection and rotation angle along the long axis (*x* axis), and *s* denotes the coordinate along the beam. Lower-left inset: modelling of a microbubble, where *R*_c_ is the radius of the cavity, *R* is the curvature radius of the trapped microbubble and *a* is the amplitude of the centre displacement during oscillation. Scale bars, 1 cm (**a**,**d**).

To demonstrate the selective excitation capability of the artificial muscle, we further investigated the deformation of an artificial muscle equipped with variable-size microbubble arrays. The muscle, measuring 3 cm × 0.5 cm × 80 μm, contains 3 arrays of microbubbles with diameters of 12 μm, 16 μm and 66 μm, each with a depth of 50 μm. Upon stimulation at its resonance frequency (96.5 kHz), the 12 μm × 50 μm microbubble array, covering an area of 0.5 cm^2^, induced a leftwards deformation in the corresponding muscle region, as depicted in Fig. 2a and Supplementary Video 3. Similarly, when the frequency was respectively increased to match the resonance frequencies of the 16-μm (82.3 kHz; Fig. 2b) and 66-μm (33.2 kHz; Fig. 2c) bubble arrays, the muscle showed a localized leftwards deformation in the middle region and bottom region, respectively. We further demonstrated an undulatory sinusoidal-like deformation by actuating the artificial muscle with a sweeping-frequency ultrasound excitation (20 kHz to 90 kHz). This continuous, time-dependent motion, as shown in Fig. 2d and Supplementary Video 4, resulted from the periodic reverse thrust generated across different regions of the muscle.

## Modelling of microbubble-array artificial muscles

We have developed a theoretical model to improve our understanding of the response of soft artificial muscles to sound waves. This model divides the entire artificial muscle into discrete segments that correspond to the patterned microbubble arrays, as illustrated in Fig. 2e. We began by modelling the acoustofluidic thrust force from a single trapped microbubble and analysing the resulting artificial muscle deformation. To formulate the model, we assumed that (1) the ultrasound produces a homogeneous oscillating pressure field at the microbubble, leading to the thrust force; (2) the beam’s oscillation amplitude is negligible compared with that of the microbubbles, such that its motion does not significantly affect the surrounding flow field; (3) hydrodynamic coupling between oscillating microbubbles can be neglected; (4) the fluid is incompressible; (5) beam stretching is negligible; and (6) the gravity of the muscle does not influence the beam deformation.

**Extended Data Fig. 3:**
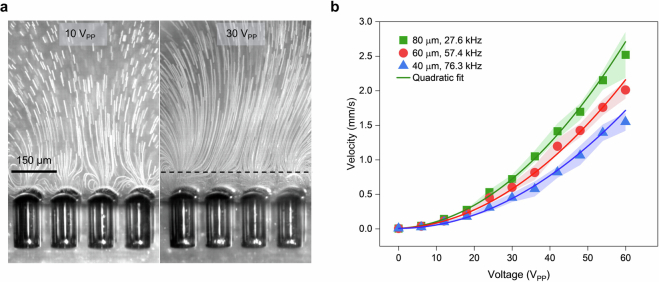
Measurement of microstreaming velocity by PIV. **a**, Microstreaming generated by a 4×4 80 μm × 150 μm microbubble array under an excitation frequency of 27.6 kHz and two different voltages of 10 V_PP_ (left panel) and 30 V_PP_ (right panel). The black dashed line denotes the measurement position of the microstreaming velocity, which is 80 μm away from the surface. **b**, Plot of microstreaming velocity versus ultrasound excitation voltage respectively measured by 4×4 microbubble arrays with three different sizes (40 μm, 60 μm, and 80 μm). The solid lines are the quadratic fitting results. The shaded error bands represent mean ± s.d. from *n* = 5 independent measurements.

To calculate the thrust force arising from acoustic streaming generated by a single oscillating microbubble, we adopted a model developed by refs. ^48,49^. With additional approximations (Supplementary Note 1), we derived an expression for the thrust force

1 $${F}_{i}\approx {\rm{\pi }}\rho \omega {{R}_{{\rm{c}}i}}^{3}{v}_{i},$$

where *ρ* is the fluid density, *ω = *2π*f* with the ultrasound frequency *f*, *R*_c*i*_ is the cavity radius in segment *i*, and *v*_*i*_ is the mean tangential velocity along the microbubble surface perpendicular to the beam, measured experimentally (Extended Data Fig. 3). For example, a microbubble with a 30 μm radius and 150 μm depth in water (*ρ* = 1,000 kg m^−3^), excited at 57.4 kHz with 60 V_PP_, produced a measured velocity of *v*_*i*_ = 2.01 mm s^−1^, yielding a thrust force of *F*_*i*_ = 61 nN according to equation (1). Scaling this to an array of approximately 18,500 uniformly sized microbubbles on a 30 mm × 5 mm artificial muscle yields a total force reaching up to 1.1 mN, corresponding to a force intensity of 7.6 μN mm^−2^ (Supplementary Fig. 4).

To describe the beam deformation, we parameterized the slender beam length by a variable *s*. Owing to planar symmetry, the deformation is fully described by the local slope angle *θ*(*s*). Using linear elasticity and the known orthogonal thrust force density, we derived the governing equation for *θ*(*s*) (Supplementary Note 2). Assuming small variations in *θ* within each segment, we obtained an analytical expression for *θ*(*s*) in terms of the beam’s Young’s modulus *E*, second moment of area *I* and the segmental thrust force densities. The resulting *y*-direction deformation as a function of *s* is then given by

2 $$\varDelta (s)={\int }_{0}^{s}\sin (\theta ({s}^{{\prime} })){\rm{d}}{s}^{{\prime} }.$$

Our model is applicable to artificial muscles featuring both uniform-size and variable-size microbubble arrays. The deformation amplitude of an artificial muscle can be amplified quadratically by increasing the ultrasound excitation voltage $(\varDelta \propto {{\rm{V}}}_{{\rm{PP}}}^{2})$, as shown in Supplementary Fig. 4. The deformation can also be increased by increasing the number of microbubbles (Supplementary Fig. 5). In addition, larger deformation can be achieved by either reducing the material’s Young’s modulus or decreasing the muscle’s thickness (Supplementary Fig. 6). Furthermore, we envision that expanding the range of microbubble sizes enhances the manipulation freedom.

## Applications of microbubble-array artificial muscles

**Fig. 3:**
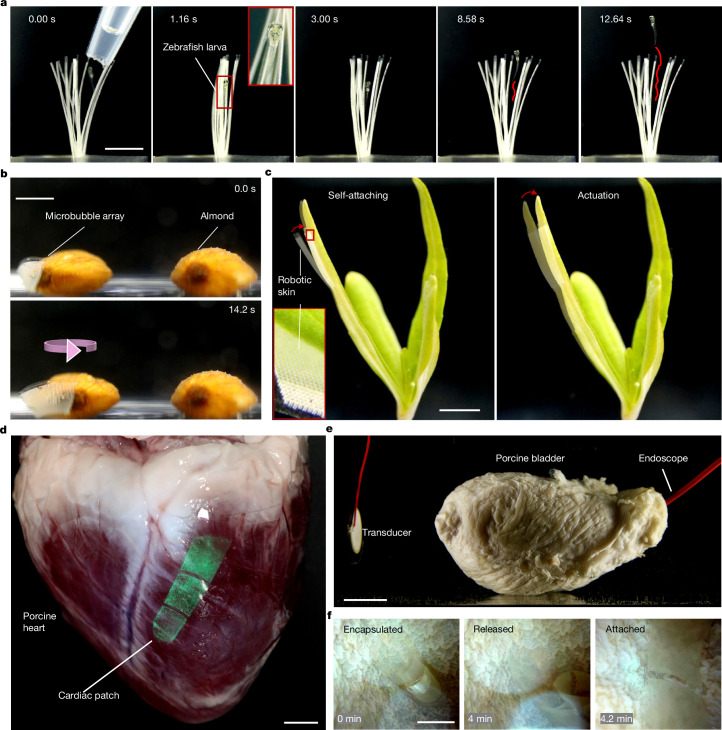
Adaptive gripper and robotic skin based on microbubble-array artificial muscles. **a**, Time-lapse sequence showing a live zebrafish larva grasped by a soft gripper composed of multiple artificial-muscle petals (10 mm × 0.7 mm × 80 µm), each incorporating microbubble arrays (12 µm in diameter × 50 µm in depth). Inset: magnified view of the larva. **b**, Rotation of an almond by a conformable microbubble-array robotic skin (12 μm × 50 μm). **c**, Deformation of a blade of grass by the same robotic skin, showing self-attachment and actuation. Inset: magnified view of the microbubble array. **d**, Conformal attachment of a green fluorescently labelled cardiac patch (30 mm × 10 mm × 80 μm) to the epicardial surface of an ex vivo porcine heart. **e**, Experimental set-up showing an excised porcine bladder with an ultrasound transducer positioned approximately 5 cm from the left side and an endoscope inserted for internal visualization. **f**, Time-lapse endoscopic images showing the encapsulated artificial muscle inside the bladder, its release at approximately 3–5 min and conformal attachment to the inner wall at 4.2 min under ultrasound activation. Scale bars, 5 mm (**a**–**c**), 1 cm (**d**,**f**), 2 cm (**e**).

The development of programmable microbubble-array artificial muscles presents an exciting alternative for wireless actuation, enabling innovative designs in the field of soft robotics. Trapping and manipulating small, fragile model animals (for example, zebrafish embryos) could become an appealing area of research in soft robotics. Conventional micro-tweezers often lack sufficient gripping force and bulkier grippers risk damaging delicate targets. To address this, we designed a miniaturized soft gripper composed of six to ten uniform-size microbubble-array artificial muscles. Each tentacle houses approximately 10,000–20,000 microbubbles when submerged in water. As illustrated in Fig. 3a and Supplementary Video 5, when subjected to an ultrasound stimulus (95.5 kHz, 60 V_PP_), the tentacles gripped a zebrafish larva within 100 ms. When the ultrasound stimulus was deactivated, the larva easily swam away (Supplementary Video 6). Repeated actuation showed no notable heating or adverse effects on the larva, confirming the biocompatibility of the mechanism.

We further demonstrated the artificial muscle as a conformable robotic skin capable of adhering to arbitrary surfaces and imparting motion to stationary objects. For example, we attached the robotic skin containing a uniform-size microbubble array to an arbitrary-shaped almond that exhibited controllable anticlockwise rotation upon excitation at 95.5 kHz and 60 V_PP_ (Fig. 3b and Supplementary Video 7). We further show that upon switching on the ultrasound excitation, the robotic skin self-adhered to a blade of grass and enabled it to bend (Fig. 3c and Supplementary Video 8). The microbubble-array robotic skin offers the inanimate object diverse mobilities without notable size or mass increase.

**Extended Data Fig. 4:**
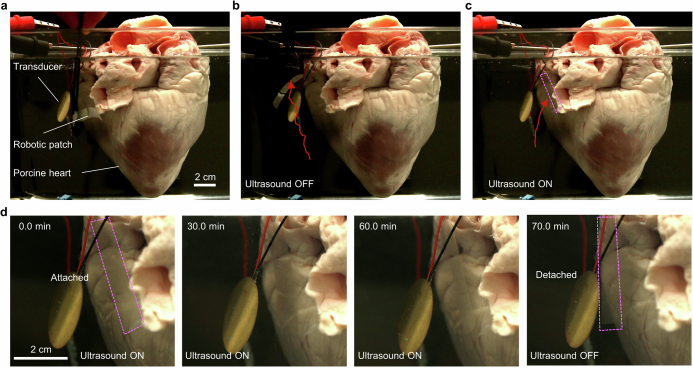
Attachment of a robotic patch to an ex vivo porcine heart. **a**, Experimental setup showing an artificial muscle positioned between an ultrasound transducer (operated at 96 kHz and 60 V_PP_) and the heart, separated by ~2.5 cm. **b**, The muscle is released from the tweezer positioned at the bottom and rises upward due to buoyancy (trajectory indicated by the red arrow). **c**, The artificial muscle conforms to the surface of the heart when stimulated by ultrasound (trajectory indicated by the red arrow). **d**, Time course of attachment robustness, demonstrating conformation and adhesion of the artificial muscle to the heart from 0 to 60 min under ultrasound actuation, followed by detachment upon ultrasound deactivation at 70 min. The pink dashed rectangle indicates the location of the artificial muscle.

**Extended Data Fig. 5:**
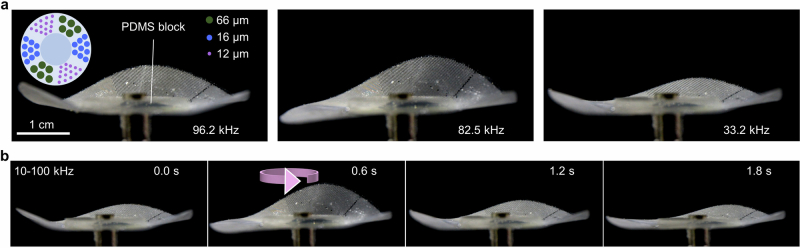
Multimodal shape transformation of a microbubble array-patterned functional surface. **a**, Multimodal shape transformation of a circular surface under continuous ultrasound excitation frequencies of 96.2 kHz, 82.5 kHz, and 33.2 kHz, respectively. The circular surface was topped with a circular PDMS block (2 cm diameter, 0.3 cm thickness) to reduce buoyancy. The inset shows a schematic of the microbubble array patterned on the surface. **b**, Dynamic shape transformation of the circular surface under sweeping-frequency ultrasound excitation spanning from 10 kHz to 100 kHz over 2 s.

**Extended Data Fig. 6:**
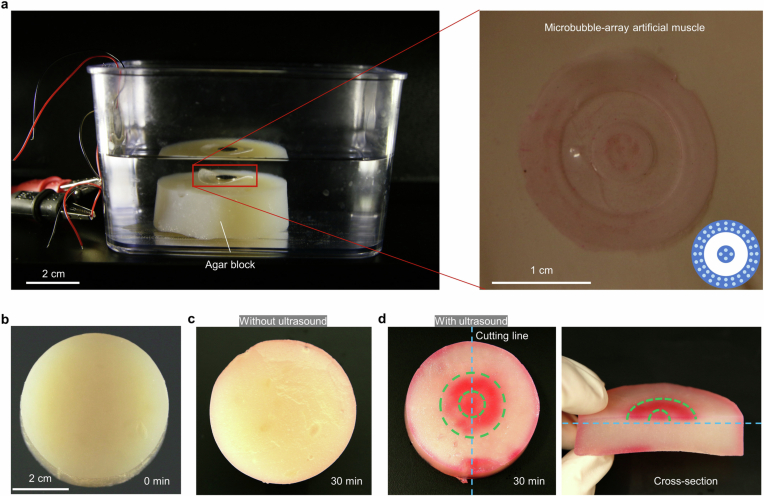
Ultrasound-enhanced dye delivery into an agar phantom by a microbubble-array robotic patch. **a**, Setup illustrates an agar block resting in an acoustic tank, with a piezo transducer positioned 5 cm to the left. A circular robotic patch is placed on the agar block with its microbubble arrays facing downward. The zoom–in highlights the patch surface; inset shows the patterned microbubble array. **b**, Agar block prior to dye exposure. **c**, Control condition showing the agar block after 30 min in a dye-filled tank without ultrasound actuation. **d**, Top and cross-sectional views of the agar block after 30 min of ultrasound actuation (96 kHz, 60 V_PP_), revealing enhanced dye penetration. The blue dashed line indicates the cutting plane; green dashed lines delineate the boundaries of the penetrated region.

Similarly, we demonstrated conformal attachment of the robotic skin—an artificial muscle containing a uniform-size microbubble array—to the epicardial surface of an ex vivo porcine heart, where it maintained functional adhesion for over 60 min at 96 kHz and 60 V_PP_ (Fig. 3d, Extended Data Fig. 4 and Supplementary Video 9). By engineering different microbubble arrays into circular geometry and tuning the excitation frequency, we generated selective and programmable localized mechanical forces, multimodal shape transformations (Extended Data Fig. 5 and Supplementary Video 10) and targeted drug delivery (Extended Data Fig. 6). Localized stimulation enables on-demand mechanical actuation of soft biological tissues and could support a range of future cardiac therapies and clinically relevant interventions, such as targeted anti-fibrotic drug delivery and localized gene or messenger RNA therapy. These results highlight the potential for the future development of in vivo wireless and wearable devices.

To evaluate the potential for wireless robotic drug delivery and in situ deployment, the artificial muscle was pre-encapsulated in a biodegradable capsule designed for swallowable or minimally invasive delivery (Fig. 3e). Upon injection into an excised porcine bladder, the capsule gradually dissolved in about 3–5 min, exposing the actuator to the surrounding environment. Following dissolution, ultrasound (96 kHz, 60 V_PP_) was applied to induce deformation of the actuator, allowing it to attach to the inner surface of the bladder (Fig. 3f and Supplementary Video 11).

**Fig. 4:**
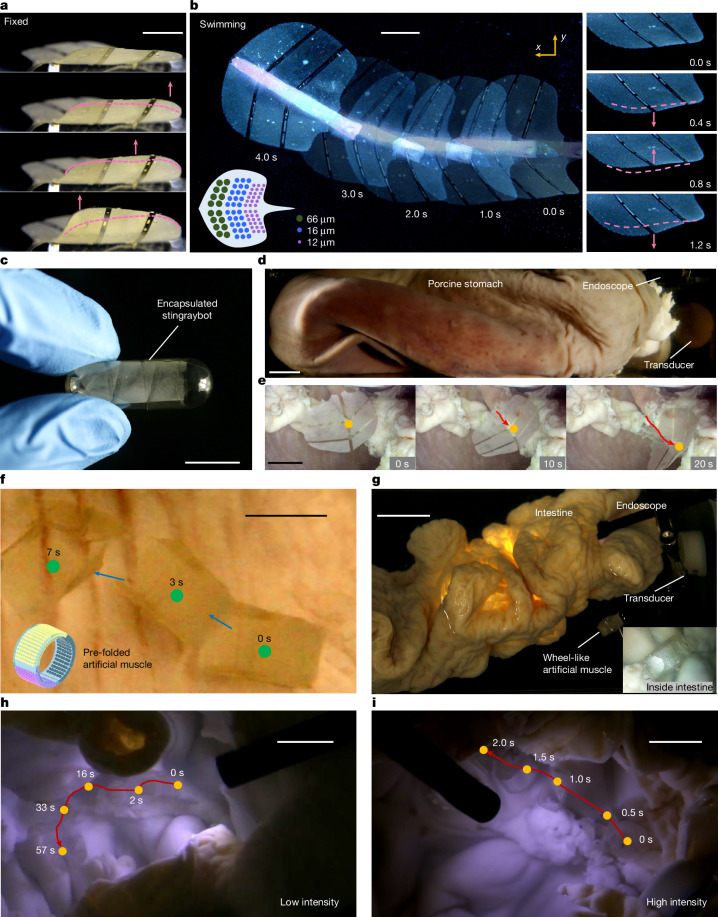
Bioinspired swimming and navigation within ex vivo biomedical environment. **a**, Undulatory motion of the microbubble-array fins (12 μm, 16 μm and 66 μm in diameter, 50 μm in depth) of the bioinspired stingraybot before release. **b**, Forward swimming of the stringraybot under sweeping-frequency excitation (30–90 kHz, 2 s, 60 V_PP_). Right: fin motion during swimming. Lower inset: schematic of the patterned microbubble arrays. In **a** and **b**, the pink dashed lines and arrows denote the fin shapes in last step and the fin’s moving direction, respectively. **c**, Edible hydroxypropyl methylcellulose capsule (27 mm × 12 mm) containing a pre-folded stingraybot. **d**, Set-up for release and navigation of the encapsulated artificial muscle in an excised porcine stomach, with an external transducer positioned approximately 3 cm from the stomach and internal endoscope for visualization. **e**, Locomotion of the stingraybot inside an excised porcine stomach. **f**, Locomotion of a pre-folded, wheel-shaped artificial muscle (30 mm × 5 mm × 80 μm) with variable-size microbubble arrays (12 μm, 16 μm and 66 μm in diameter, 50 μm in depth) inside a porcine stomach. The artificial muscle propels along the stomach surface under sweeping-frequency excitation (30–100 kHz, 2-s sweep period, 60 V_PP_). The blue arrows mark the direction of motion and the green dots indicate the centre position. Inset: pre-folded shape. **g**, Set-up for ex vivo manipulation of a pre-folded artificial muscle inside an excised porcine intestine, with external transducers and an internal endoscope. Inset: endoscopic view of the artificial muscle. **h**, Time-lapse images showing the artificial muscle rolling along the curved mucosal wall under ultrasound sweeping-frequency (30–100 kHz, 2-s sweep period, 60 V_PP_) delivered by a piezo transducer. **i**, Locomotion of the artificial muscle driven by a high-intensity focused ultrasound transducer (1–3 MHz, 1-s sweep period, 60 V_PP_). Red lines, trajectory; yellow dots, centre position over time. Scale bars, 1 cm (**a**–**c**,**e**,**f**,**h**,**i**), 2 cm (**d**,**g**).

Capitalizing on the dynamic deformation and rapid response capabilities of our artificial muscle, we engineered a bioinspired ultrasound-powered wireless stingraybot. The biomimetic stingraybot features two artificial muscles—designed to mimic the pectoral fins of a natural stingray—integrated on its sides. These pectoral fins incorporate arrays of differently sized microbubbles (12 μm, 16 μm and 66 μm in diameter, 50 μm in depth) patterned along the head-to-tail axis and paired with a PDMS block for buoyancy adjustment. When exposed to a sweeping-frequency ultrasound stimulation (30–90 kHz over 2 s at 60 V_PP_), the stingraybot’s fins exhibit an undulatory motion that mimics the natural motion of a stingray (Fig. 4a and Supplementary Video 12). Upon release, the stingraybot propels forward at an initial speed of about 0.8 body lengths per second (Fig. 4b). More control experiments on stingraybot propulsion are provided in Methods.

To implement practical biomedical applications, we demonstrated ultrasound-guided navigation of a pre-folded artificial muscle through ex vivo porcine gastrointestinal tissues, targeting use cases such as site-specific drug release for gastrointestinal disorders, minimally invasive access to inflamed or fibrotic tissue, and wireless actuation in regions inaccessible to rigid tools. We first pre-folded and encapsulated a stingraybot within an edible capsule (Fig. 4c). Once released into the stomach (Fig. 4d), the stingraybot propelled on demand within the confined biomedical environment under ultrasound actuation (Fig. 4e and Supplementary Video 13). In a separate experiment, we pre-folded a linear artificial muscle—with variable-size microbubble arrays arranged along its outer surface—into a cylindrical, wheel-like structure. Under sweeping frequencies, the actuator exhibited directional rolling propulsion along the complex mucosal surfaces of the stomach and intestine (Fig. 4f–i and Supplementary Video 14), illustrating its potential for soft robotic intervention and targeted delivery within the gastrointestinal tract. Future work will focus on parametric studies, dynamic folding strategies and steering-enabled configurations of the artificial muscle across varied tissue geometries and fluidic environments.

## Discussion

We have introduced a class of soft artificial muscles that use acoustically activated microbubble arrays to achieve programmable actuation. These artificial muscles show dynamic programmability, high force intensity (about 7.6 μN mm^−2^), rapid responsiveness (sub-100 ms) and wireless controllability, all while maintaining exceptionally compact (3,000 microbubbles per mm^2^) and lightweight (0.047 mg mm^−2^). Through the strategic use of microbubble configurations and voltage and frequency as ultrasound excitation parameters, we engineered a diverse range of preprogrammed movements (for example, undulatory motion) and demonstrated their applicability across various robotic platforms. We showcased the strength and durability of these muscles by integrating variable-size microbubble arrays into functional devices such as a soft gripper, a robotic skin and a biomimetic stingray robot. We also established a theoretical model that elucidates the actuation mechanism, which serves as a guide for the design of microbubble-array patterns with enhanced actuation performance. These artificial muscles offer extensive applications in robotics, flexible electronics, wearable technologies, prosthetics, biomedical instrumentation and beyond.

To optimize the artificial muscle performance through the geometric design and the density of the microcavities, preliminary experiments revealed that converging trapezoidal cavities generate roughly three-times-stronger streaming velocities than diverging shapes (Supplementary Fig. 7) and a higher density of cavities causes a larger deformation. By incorporating geometric and density optimization with systematic characterization, one can establish a predictive design framework for actuators with tailored deformation profiles—enabling precise control in applications from soft robotics to biomedical devices. Future studies could also explore the application of confocal sound sources, such as high-intensity focused ultrasound (Supplementary Fig. 8) to achieve local millimetre deformation—which potentially could lead to tools for applications such as in vivo mechanotransduction and spatially targeted drug delivery. In addition, the bubble-based mechanism is widely material agnostic and can be extended to biocompatible or biodegradable matrices, such as hydrogels and biodegradable polymers for more biomedical applications. More robustness evaluations on our ansatz across fluid media are provided in Methods.

Despite promising results, certain limitations remain. Prolonged actuation triggers microbubble growth within the cavities, destabilizing the muscle operation after approximately 30 minutes (Supplementary Fig. 9). Resubmersion in water restores the function, and sealing cavities with a thin PDMS membrane will offer a long-term robust solution (Supplementary Fig. 10). In addition, the stingraybot’s distance-dependent actuation must be taken into account for untethered operation. Our preliminary experiments at varying transducer distances revealed deformation decays with increasing distance (Supplementary Fig. 11), dropping by about 50% at 5 cm compared with the deformation at 1 cm. Although this limitation is less critical in vivo (where the robot is intended to operate in confined volumes, for example, the bladder), optimizing the ultrasound source configurations and the actuation voltage to compensate for ultrasound intensity decay over distance can enhance the performance.

**Extended Data Fig. 7:**
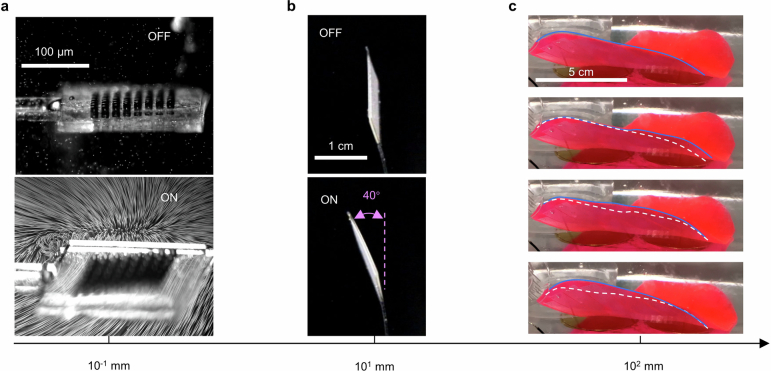
Artificial muscles functioning at scales from 10^−1 ^mm to 10^2 ^mm. **a**, A microscale rotator featuring an asymmetric 8 × 8 microbubble array (12 μm × 50 μm). The upper and lower panels show the microrotator with ultrasound off and on, respectively, at 95.5 kHz and 60 V_PP_. *n* = 3 independent measurements. **b**, A millimeter-scale artificial muscle with an asymmetric 400 × 200 microbubble array (12 μm × 50 μm). The upper and lower panels show the device with ultrasound off and on, respectively, under the same driving conditions. The purple dashed line indicates the original position of the artificial muscle. **c**, A macroscale stingraybot equipped with artificial muscles comprising variable-size microbubble arrays (40 μm × 150 μm, 60 μm × 150 μm, 80 μm × 150 μm, respectively), demonstrating undulatory motion under excitation (10–90 kHz, duty cycles 2 s, 120 V_PP_). The blue line marks the current location of the fin edge, while the white dashed line shows its position in the previous frame of the time-lapse image.

Looking ahead, these artificial muscles hold transformative potential across cutting-edge fields such as soft robotics, haptic medical devices and minimally invasive surgery. Future research should focus on refining the scalability of these systems across multiple scales (Extended Data Fig. 7), enhancing their force-generation capabilities and integrating them into complex devices for biomedical applications.

## Methods

### Fabrication of artificial muscles

The negative patterns of the artificial muscles were first designed in commercial electronic design automation software (as shown in Supplementary Fig. 1). The patterns were transferred into a photomask by a direct writing laser (DWL2000) machine in a clean room (BRNC). Then we spin-coated the negative photoresist SU8-3025 on a 4-inch silicon wafer. Using standard lithographic fabrication, the patterns were transferred to the photoresist via exposure to ultraviolet light through the mask. After the developing process, the negative patterns of the microbubble arrays, that is, micropillars, were additive on the wafer. The height of the micropillar depends on the spinning speed. Next, to enhance the surface properties, a silane-based hydrophobic treatment was applied to the 4-inch wafer with micropillars for 1 h (see fabrication flow in Supplementary Fig. 12). The PDMS used in this process was prepared with a 10:1 ratio of the base to curing agent. Then the PDMS mixture was poured onto the wafer. To ensure a high-quality coating, the mixture was degassed under a vacuum pressure of less than 1 mbar. After degassing, spin-coating of the PDMS was performed on the wafer. Different spin speeds resulted in varying PDMS membrane thicknesses (Supplementary Fig. 2). After spin-coating, the PDMS was vacuumed again and cured in a sequential heating process: 1 h at 60 °C, followed by 1 h at 80 °C and finally 1 h at 100 °C. Finally, the PDMS soft membrane was cured and then peeled off the wafer. This process yielded a uniform PDMS layer suitable for use in artificial muscle and soft robotic applications. In all our experiments, each cavity consistently trapped only a single bubble as the artificial muscle submerged into the water (Supplementary Fig. 13).

### Acoustic set-up

For the microscale characterization of microbubbles, the experimental set-up was built on a thin glass substrate with dimensions of 24 mm × 60 mm × 0.18 mm. As shown in Supplementary Fig. 14, a circular piezoelectric transducer (27 mm × 0.54 mm, resonance frequency 4.6 kHz ± 4%, Murata 7BB-27-4L0) was affixed to the glass substrate using an epoxy resin (2-K-Epoxidkleber, UHU Schnellfest). A square PDMS acoustic chamber (10 mm × 10 mm × 5 mm) was positioned in front of the transducer, which was filled with deionized water and covered with a cover glass (22 mm × 22 mm × 0.18 mm). An artificial muscle was suspended in the centre of the chamber with one end clamped to the side wall and the other end left free. The substrate was then mounted on an inverted microscope (Axiovert 200M, ZEISS).

For the macroscale actuation of artificial muscles by sound, the experimental set-up consisted of a plastic tank measuring 10 cm × 10 cm × 8 cm with a wall thickness of 2 mm. For ex vivo porcine experiments, a larger chamber (30 cm × 15 cm × 15 cm, thickness 2 mm) was used. As shown in Supplementary Fig. 15, the circular piezoelectric transducers were affixed to the inside surfaces and the bottom surface of the tank using the epoxy resin or directly submerged into the liquid. An artificial muscle was suspended inside the chamber with one end clamped, and three cameras were placed around the tank to capture the actuation of acoustic artificial muscles from multiple viewing angles. In addition, a miniaturized endoscopic camera (8 mm diameter and 1080P resolution, FuanTech) was used to capture images inside the porcine specimens. An electronic function generator (AFG-3011C, Tektronix) and an amplifier (0–60 V_PP_, ×15 amplification, High Wave 3.2, Digitum-Elektronik) were connected to the transducer to generate sound waves with tunable excitation frequencies and voltages. Square waves effectively drive the artificial muscle, achieving maximum deformation and outperforming other tested waveforms, such as sinusoidal and triangular waveforms under equivalent excitation conditions (Supplementary Fig. 16).

### Microstreaming characterization

We evaluated the microstreaming jets generated by ultrasound-driven microbubbles embedded in the muscle using 6-μm tracer particles in water and particle image velocimetry analysis. Three uniform-size microbubble arrays, each comprising a 4 × 4 grid of microbubbles with diameters of 40 μm, 60 μm and 80 μm (150 μm in depth), were individually selected and tested in separate miniaturized artificial muscles (500 μm × 500 μm × 200 μm; Extended Data Fig. 3a and Supplementary Video 15). When activated at their respective resonance frequencies 76.3 kHz, 57.4 kHz and 27.6 kHz, we measured the microstreaming velocity 80 μm away from the bubble interface and observed a quadratic relationship between the average velocity and the excitation voltage (Extended Data Fig. 3b). The streaming velocity near the bubble reached 2.5 mm s^−1^ at 60 V_PP_. This voltage-dependent microstreaming directly correlates with the reverse thrust generated by the microbubble array, demonstrating that the thrust magnitude can be dynamically tuned by adjusting ultrasound excitation.

We further investigated the selective actuation of a variable-size microbubble array of 40 μm, 60 μm and 80 μm diameter, each 150 μm in depth, integrated within a single miniaturized artificial muscle (500 μm × 500 μm × 200 μm) with corresponding frequencies (76.3 kHz, 57.4 kHz and 27.6 kHz, respectively). The particle image velocimetry analysis revealed that the microstreaming developed by the 80-μm bubbles generated an average velocity of 0.23 mm s^−1^ at 27.6 kHz, which was markedly stronger compared with the velocities (<0.05 mm s^−1^) produced by the other two microbubble arrays at the same voltage (15 V_PP_). Similarly, adjusting the frequency to 57.4 kHz (76.3 kHz) selectively activates the 60 μm (40 μm) bubble array, resulting in more intense streaming at 0.174 mm s^−1^ (0.075 mm s^−1^), in contrast to other arrays (Extended Data Fig. 2). Additionally, applying a sweeping frequency (10–90 kHz) over 4 s at 30 V_PP_ enabled wave propagation across the artificial muscle (Supplementary Video 16).

### Control experiments on artificial muscle deformation

To determine the key factors influencing muscle deformation, a set of control experiments was performed. We first examined the streaming jets of a uniform-size microbubble-array artificial muscle (1 cm × 0.3 cm × 80 μm) patterned with over 800 microcavities (each 40 μm in diameter and 50 μm in depth). Supplementary Video 17 shows that an artificial muscle without microbubbles exhibited minor deformation, with no noticeable microstreaming observable across the excitation frequency sweeps from 1 kHz to 100 kHz at 60 V_PP_. By contrast, the actuator exhibited pronounced deformation at an excitation frequency as low as 9.5 kHz (well below resonance), where microbubbles generated microstreaming (approximately 0.8 mm s^−1^), resulting in substantially greater deformation compared with the case without microbubbles.

### Repeatability and characterization of artificial muscle deformation

**Extended Data Fig. 8:**
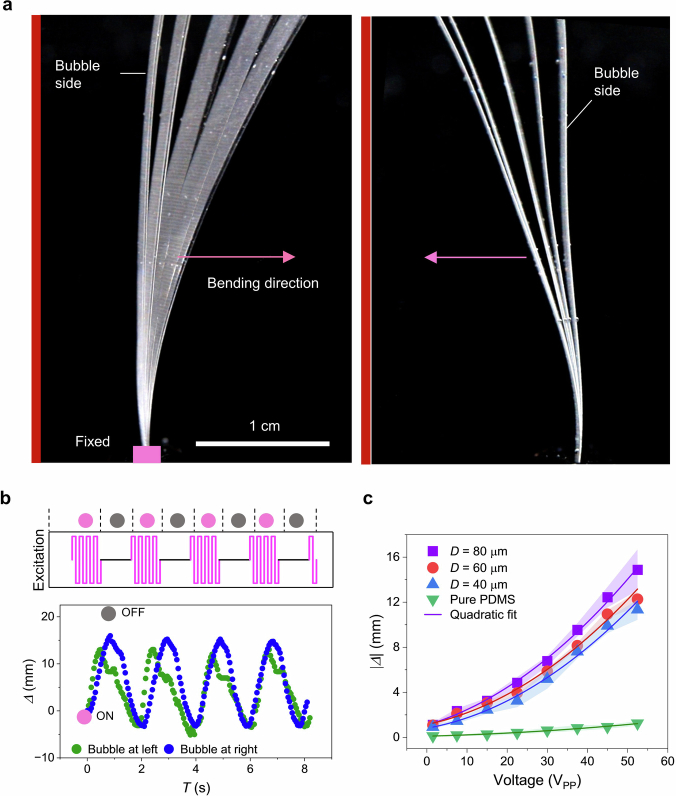
Deformation of uniform-size microbubble array artificial muscle. **a**, Time-lapse images of a uniform-size microbubble array artificial muscle with microbubbles respectively positioned at the left and right side. The pink rectangle and arrow show the fixed end and bending direction of the muscle, respectively. The red line on the left side denotes the location of the transducer. **b**, Plot of the bending amplitude of the muscle tip over multiple excitation cycles with an average repeated-positioning error of ±0.8 mm with the excitation signal shown in the top panel. The pink and gray dots correspond to the excitation ‘on’ (80.5 kHz and 52.5 V_PP_) and ‘off’ stages, respectively. The green and blue dots represent the measured deformation amplitudes when the bubbles are positioned on the left and right sides, respectively, as shown in **a**. **c**, Bending amplitude of uniform-size microbubble array artificial muscles with microbubble diameters (*D*) of 40 μm, 60 μm, and 80 μm and without microbubbles under excitation voltages from 1.5 to 52.5 V_PP_. The microbubbles have a constant depth of 50 μm. The dots and solid lines are the experimental results and quadratic fitting results, respectively. The shaded error bands represent mean ± s.d. from *n* = 5 independent measurements. All the muscles bent in the direction as shown in the left panel of **a**.

We assessed the repeatability of the artificial muscle’s deformation under identical excitation conditions, with the transducer close to the microbubble-embedded side, as shown in the left panel of Extended Data Fig. 8a. When stimulated with ultrasound pulses (80.5 kHz, 52.5 V_PP_ and 1-s on/off cycle), the muscle exhibited repeatable bending within 150 cycles, with an error of ±0.8 mm, representing 2.7% of the total beam length (Extended Data Fig. 8b). With more excitation cycles (500 cycles) of the artificial muscle, the deformation exhibited larger error (about 10%). After 10,000 cycles, there were no observable microbubbles in the artificial muscle, and the artificial muscle showed minor deformation. Furthermore, Extended Data Fig. 8c shows a quadratic relationship between the applied voltage and the mean deformation amplitude of artificial muscles, each patterned with uniformly sized microbubbles of 40 μm, 60 μm or 80 μm, when driven at their respective resonance frequencies (80.5 kHz, 62.5 kHz and 30.3 kHz). In addition, the PDMS beam, in the absence of microbubbles, exhibited limited bending (about 7% of the 40-μm microbubble-array artificial muscle’s deformation at 52.5 V_PP_) caused by the weak radiation force from incident sound waves originating from the transducer.

### Control experiments on stingraybot propulsion

In control experiments, a stingraybot without microbubbles exhibited no undulatory motion along its fins under ultrasound excitation and sank without notable lateral displacement (Supplementary Video 18). Notably, under continuous excitation at a single frequency (tested separately at 33.2 kHz, 85.2 kHz and 96.2 kHz at 60 V_PP_), targeting microbubble arrays with cavity diameters of 66 μm, 16 μm and 12 μm, respectively, the stingraybot exhibited only limited locomotion (<1 body length). By comparison, sweeping-frequency excitation (10–100 kHz over 2 s) elicited sustained undulatory motion, allowing the stingraybot to swim a significantly greater distance (>3.5 body lengths), as shown in Supplementary Fig. 17. These results suggest that the forward motion of the stingraybot is dominated by the propulsion force generated by the sequential undulatory motion, resulting from the reverse thrust generated by the microbubble arrays. Moreover, enhancing the design of the stingraybot with additional microbubble sizes could expand its manoeuvrability. For instance, integrating a navigation tail with microbubble arrays of different sizes on either side enables directional control. When activated at their respective resonance frequencies on one side, these arrays generate an asymmetric torque (Supplementary Fig. 18), enabling steering of the stingraybot via tail rotation. As the stingraybot is stealthy and transparent, we further envision that our stingraybot could be used for environmental data collection or behavioural research on real organisms, for example, detecting water quality within coral reefs and recording swarm interaction by blending into schools of fish.

### Robustness evaluation

**Extended Data Fig. 9:**
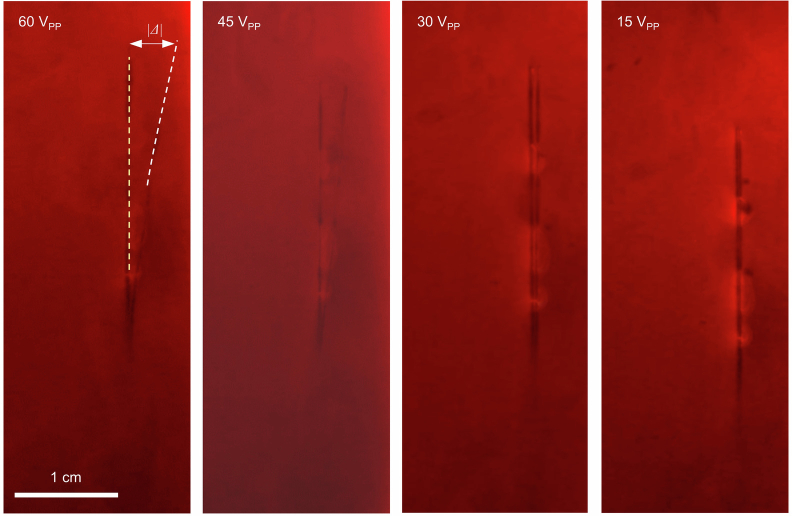
Ultrasound-induced deformation of an artificial muscle in porcine blood. An artificial muscle embedded with uniform microbubbles (12 μm × 50 μm) was immersed in 100% porcine blood, with a piezoelectric transducer bonded to the bottom of the acoustic tank and positioned 3 cm from the muscle. Final deformation (|*Δ*|) of the artificial muscle as a function of excitation amplitude—0.44 cm, 0.27 cm, 0.10 cm and 0.04 cm corresponding to 60 V_PP_, 45 V_PP_, 30 V_PP_ and 15 V_PP_, respectively—under excitation at 96 kHz. Yellow dashed lines indicate the initial muscle position; white dashed lines indicate the deformed position.

**Extended Data Fig. 10:**
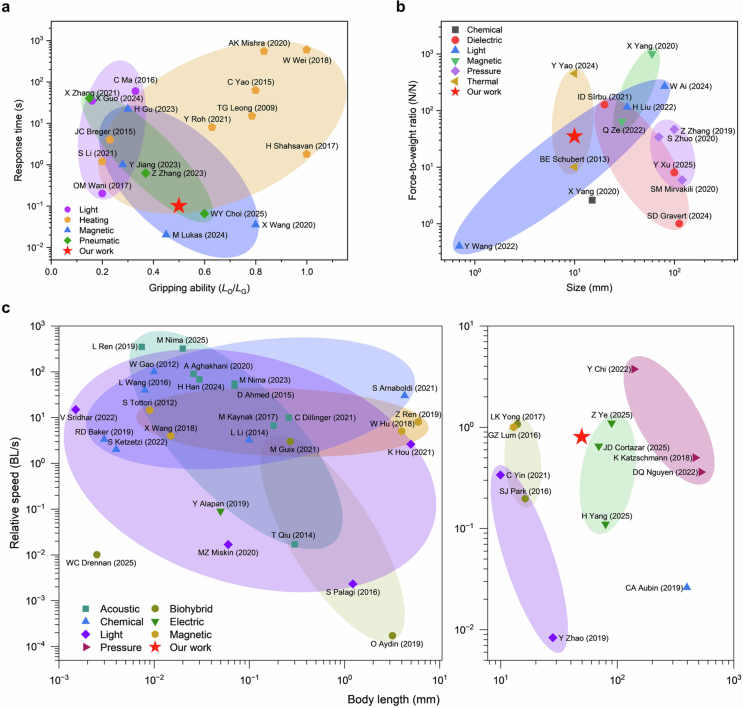
Artificial muscles performance comparison. **a**, Response time versus gripping ability (*L*_O_/*L*_G_) for grippers using different actuation methods, where *L*_O_ and *L*_G_ are the dimensions of the gripped object and gripper, respectively. **b**, Comparison of force-to-weight ratios of grippers versus their size for various actuation methods. **c**, Comparison of relative swimming speeds (body lengths per second) of swimmers with different actuation mechanisms across various scales (microscale to macroscale). Shaded regions (convex hulls) indicate typical performance ranges; representative studies are labeled by author and year.

To evaluate the robustness of our ansatz across fluid media, we quantified artificial muscle deformation in 100% porcine blood, observing amplitudes of approximately 0.4 mm, 1.0 mm, 2.7 mm and 4.4 mm at 15 V_PP_, 30 V_PP_, 45 V_PP_ and 60 V_PP_, respectively, under 96-kHz ultrasound excitation (Extended Data Fig. 9). As complementary evidence, we studied the artificial muscle performance in various aqueous solutions (deionized water, tap water and 25–100% glycerol solutions) as shown in Supplementary Fig. 19. The deformation showed an inverse relationship with glycerol concentration, with the largest deformation of about 11.3 mm in a 25% glycerol solution, followed by about 8.4 mm in 50% glycerol and 3.7 mm in 75% glycerol. The deformation was almost negligible in 100% glycerol. These results clearly demonstrate that the actuator functions effectively in full blood, validating its potential for in vivo applications in fluids with physiological viscosity. We next evaluated artificial muscle actuation in the presence of solid obstructions (Supplementary Fig. 20). A frontal obstruction (partially blocking ultrasound) reduced the deformation by 80–90% (0.5–1-mm tip deformation versus 4.8 mm unobstructed). A lateral placement caused moderate attenuation (about 2.5 mm) and posterior positioning retained a better performance (3.8 mm). Furthermore, experimental results showed significant deformation of the artificial muscle behind excised porcine ribs (Supplementary Fig. 21). Thus, actuators remained functional near obstacles but required strategic positioning to maximize deformation. Our preliminary results also revealed negligible heating effects near the piezoelectric transducer during artificial muscle and stingraybot operation (Supplementary Fig. 22), underscoring the thermally benign nature of our acoustic platform. Although frequency-dependent selectivity was achieved, some cross-excitation between microbubble arrays was observed. This effect was mitigated under sweeping-frequency actuation, and temporal control over the sweep dynamics has a key role in preserving spatial selectivity and ensuring reliable, programmable motion. In vivo biomedical environments present additional challenges such as complex fluid flow, irregular geometry and variable temperature gradients, all of which may distort ultrasound propagation. Although the actuator showed robust and competitive performance under static conditions with other methodologies (Extended Data Fig. 10 and Supplementary Fig. 23), future work will explore flow-resilient designs, including optimized microbubble-array geometries, flexible ultrasound configurations and real-time actuation control strategies to maintain reliable performance in dynamic fluid environments.

### Numerical simulations

Finite element numerical simulations were conducted using the commercial COMSOL Multiphysics software (v6.1), including simulations on the acoustic pressure field in the small PDMS chamber, acoustic streaming generated by variable-size microbubbles in the small PDMS chamber, the acoustic pressure field in the big acoustic tank and the deformations of the artificial muscle. All simulations were performed with dimensions and material properties consistent with the experiments. Physics modules of simulations on acoustic pressure include solid mechanics, electrostatics, pressure acoustics fields, creeping flow, and heat transfer in solids and fluids. Simulations on the deformations of artificial muscles were performed using the solid mechanics module with corresponding boundary conditions and force conditions. The microstreaming-generated thrust was assumed to be a point force that is loaded on the bottom of each microcavity. In addition, numerical calculations based on the theoretical model were performed using the commercial Matlab software (version R2021b). See Supplementary Notes for simulation details.

### Imaging and analysis

The microscale characterization of microbubbles was recorded with a high-speed camera (Chronos 1.4, Kron Technologies) attached to the inverted microscope. Recording frame rates ranged from 1,069 to 32,668 frames per second. The macroscale motion of ultrasound artificial muscles was recorded with a high-sensitivity camera (Canon 6D and 24–70-mm camera lens, Canon). The recording frame rate was 50 frames per second. Recorded footage was analysed in ImageJ. Statistical analyses were conducted using MATLAB (version R2021b), Originlab (version Origin 2023) and Excel (version 16.54).

### Preparation of the zebrafish embryo

Zebrafish (*Danio rerio*) embryos from pairwise crosses of WIK wild-type fish were raised in E3 medium (5 mM NaCl, 0.17 mM KCl, 0.33 mM CaCl_2_, 0.33 mM MgSO_4_) at 28 °C under a 14:10 h light/dark cycle. Experiments up to 5 days post fertilization are not subject to animal welfare regulations. All husbandry and housing procedures were approved by the local authority (Kantonales Veterinäramt, TV4206).

### Preparation of the porcine organs

Porcine hearts, stomachs, intestines, ribs and blood were obtained from a licensed abattoir. As the study involved only ex vivo tissues from animals slaughtered for food production, no ethical approval was required.

### Reporting summary

Further information on research design is available in the Nature Portfolio Reporting Summary linked to this article.

## Online content

Any methods, additional references, Nature Portfolio reporting summaries, source data, extended data, supplementary information, acknowledgements, peer review information; details of author contributions and competing interests; and statements of data and code availability are available at 10.1038/s41586-025-09650-3.

## Supplementary information

Supplementary Information Supplementary Notes, Figs. 1–26, Tables 1–3, legends for Videos 1–18 and References.

Reporting Summary

Supplementary Videos Supplementary Videos 1–18.

## Data Availability

The datasets that support the findings of this study are available within the paper. Correspondence and requests for materials should be addressed to the corresponding author. The time frame for response is 12 months.
