# Supplementary material for: Ultrasound-driven programmable artificial muscles
Source: Nature. 2025 Oct 29;646(8087):1096–104. doi: 10.1038/s41586-025-09650-3 (PMC12571877; doi:10.1038/s41586-025-09650-3)
Supplement: Supplementary file 1 — Supplementary Notes, Figs. 1–26, Tables 1–3, legends for Videos 1–18 and References. [file 41586_2025_9650_MOESM1_ESM.pdf]

nature portfolio

https://doi.org/10.1038/s41586-025-09650-3

Supplementary information

Ultrasound-driven programmable artificial muscles

In the format provided by the
authors and unedited

Supplementary Materials for

Ultrasound-Driven Programmable Artificial Muscles

Zhan Shi†, Zhiyuan Zhang†, Justus Schnermann, Stephan C. F. Neuhauss, Nitesh Nama, Raphael Wittkowski, Daniel Ahmed*

Corresponding author: dahmed@ethz.ch

The PDF file includes:

Supplementary Notes
Supplementary Figures 1 to 26
Supplementary Tables 1 to 3
Legends for Supplementary Videos 1 to 18
Supplementary References

Other Supplementary Materials for this manuscript include the following:

Supplementary Videos 1 to 18

Table of Contents

Supplementary Notes

Supplementary Fig. 1 | Design of artificial muscles and stingraybot.

Supplementary Fig. 2 | Artificial muscle thickness versus PDMS spin coating speed.

Supplementary Fig. 3 | Deformations of a uniform-size microbubble array artificial muscle with various transducer positions.

Supplementary Fig. 4 | Calculated force intensities.

Supplementary Fig. 5 | Deformation ($|\Delta|$) of a uniform-size microbubble array artificial muscle with varying bubble spacings.

Supplementary Fig. 6 | Deformation of a variable-size microbubble array artificial muscle under continuous and sweeping-frequency ultrasound excitation.

Supplementary Fig. 7 | Measured maximum streaming for three different bubble shapes.

Supplementary Fig. 8 | Deformation of a uniform-size microbubble array artificial muscle with confocal ultrasound excitation.

Supplementary Fig. 9 | Robustness analysis of the microbubbles at different time scales under sweeping-frequency ultrasound excitation.

Supplementary Fig. 10 | Experimental setup and streaming of membrane-sealed bubbles.

Supplementary Fig. 11 | Deformation ($|\Delta|$) of a uniform-size microbubble array artificial muscle under different excitation distances (D).

Supplementary Fig. 12 | Fabrication process of the soft artificial muscle.

Supplementary Fig. 13 | Microscopic top and side views of microbubble arrays.

Supplementary Fig. 14 | Experimental setup for microscope characterization of microbubble arrays and corresponding acoustic pressure field simulation.

Supplementary Fig. 15 | Experimental setup for artificial muscle actuation and corresponding acoustic pressure field isosurface simulation.

Supplementary Fig. 16 | Deformation of the artificial muscle under different waveform inputs as a function of applied voltage.

Supplementary Fig. 17 | Swimming distances of a stingraybot under different excitation conditions.

Supplementary Fig. 18 | Deformation of a robotic shark tail with the uniform-size microbubble array artificial muscle.

Supplementary Fig. 19 | Deformation of an artificial muscle in glycerol-water mixtures with different mixing ratios.

Supplementary Fig. 20 | Effect of surrounding solid media on artificial muscle deformation under ultrasound excitation.

Supplementary Fig. 21 | Actuation of the artificial muscle behind porcine ribs.

Supplementary Fig. 22 | Temperature variation for different ultrasound frequencies versus time.

Supplementary Fig. 23 | A microbubble-array soft gripper capturing a 35 times heavier PDMS block.

Supplementary Fig. 24 | Modeling of the uniform-size microbubble array artificial muscle.

Supplementary Fig. 25 | Deformation of an artificial muscle under ultrasound excitation.

Supplementary Fig. 26 | Comparison of artificial muscle deformation between experimental results and analytical calculations.

Supplementary Table 1 | Comparison data of the response time and gripping ability of grippers for various actuation mechanisms.

Supplementary Table 2 | Comparison data of force-to-weight ratios of artificial muscles versus their sizes for various actuation mechanisms.

Supplementary Table 3 | Comparison data of relative swimming speeds of swimmers relative to their body length for various actuation mechanisms.

Legends for Supplementary Videos

Supplementary References

Supplementary Notes

Supplementary Note 1. Thrust force developed by a single oscillating microbubble

In this work, our primary goal is focusing on the thrust force generated by oscillating microbubbles, omitting other complex interactions[S1-S4]. Experimental results confirm that thrust is the dominant actuation force driving the artificial muscle. Considering an axisymmetric microbubble trapped in a PDMS cavity (cylindrical cavity with radius R_c and depth h, as shown in the inset of **Supplementary Fig. 24**) and surrounded by fluid with the mass density ρ, the resonance frequency of the microbubble is estimated as $f_0 = \frac{1}{2\pi}\sqrt{\frac{3\pi\gamma P_0}{8R_c h\rho}}$ [45], where γ is the adiabatic index (1.4 for air) and P_0 is the atmospheric pressure. Estimating the resonance frequency of the microbubble aids in the design and measurement of its resonance characteristics.

For an analytical description of the force generated by a single bubble, we present the thrust force's scaling and show how to estimate the force given the surface streaming velocity. For this purpose, we apply the theoretical framework developed by Spelman and Lauga[48,49]. We assume incompressibility of the water and $\epsilon \ll \delta \ll 1$. Here, ϵ is the relative bubble oscillation amplitude $\epsilon = a/R$, for the bubble's (center) displacement a and curvature radius R, and $\delta = (2\eta/\omega)^{1/2}/R$ is the relative viscous penetration length with the kinematic viscosity η of the liquid and the ultrasound's (angular) frequency ω.

The incompressibility assumption implies an infinite speed of sound, which leads to neglection of the radiation force that results from the momentum transfer of the sound wave to the bubble when the sound wave is scattered at the bubble. This assumption is necessary to obtain an analytical result for the thrust force and justified because the force corresponding to the acoustic streaming is dominant compared to this radiation force – although they can be of the same order of magnitude as can be seen in **Supplementary Fig. 3**. While the assumption $\epsilon \ll \delta \ll 1$ usually holds, it is violated if ϵ is too large, which can be the case when the ultrasound frequency approaches the resonance frequency. For describing an oscillating bubble on a beam, Spelman and Lauga's work[48,49] gives the force on the beam as

$$F = \alpha\rho R^4\omega^2\epsilon^2, \tag{S1}$$

where α is a purely microbubble-oscillation-shape-dependent prefactor, and $\omega = 2\pi f$ with the excitation frequency f. ϵ depends on the incoming ultrasound frequency and intensity as well as on the microbubble oscillation shape. Since the exact value of α is unknown in our case, we calculate F using some approximations.

First of all, we deviate from our model sketched in the inset of **Supplementary Fig. 24** and bend the PDMS-water surface so that the total water surface is a sphere of the same curvature radius as the (resting) bubble. At the end of our calculation, we will further assume the bubble radius to be large, thereby absorbing the error of the assumption made here.

A non-dimensionalization is carried out canonically using $1/\omega$ as the time scale and R as the length scale. We describe the oscillating surface of the sphere by a radial coordinate r and an angular coordinate Θ (see **Supplementary Fig. 24**). As the (resting) sphere's azimuthal angle θ is used as a bubble-surface coordinate, the deformation of the bubble in radial and angular direction is time-locally given as

$$r(\theta) = 1 + \epsilon\,\mathrm{Re}[r^{(1)}(\theta)\,e^{i(t+\pi/2)}] + \epsilon^2 r^{(2)}(\theta) f(t) + O(\epsilon^3), \tag{S2a}$$

$$\Theta(\theta) = \theta + \epsilon\,\mathrm{Re}[\Theta^{(1)}(\theta)\,\mathrm{e}^{\mathrm{i}(t+\pi/2)}] + \epsilon^2\Theta^{(2)}(\theta)\,g(t) + O(\epsilon^3), \tag{S2b}$$

where $f(t)$, $g(t) = t$ plus some oscillation that vanishes under time-averaging. Further following Spelman and Lauga, an expansion of $\Theta^{(2)}$ is given by

$$\Theta^{(2)}(\mu) = \sum_{n=1}^{\infty} \alpha_n \frac{\int_\mu^1 P_n(x)\,\mathrm{d}x}{\sqrt{1-\mu^2}}, \tag{S3}$$

where P_n is the n-th Legendre polynomial and $\mu=\cos(\theta)$ is the height relative to the center of curvature. Due to the orthogonality of the Legendre polynomials, the expansion coefficients are given by

$$\alpha_n = -\frac{2n+1}{2}\int_{-1}^{1} P_n(\mu)\frac{\mathrm{d}}{\mathrm{d}\mu}\left(\sqrt{1-\mu^2}\,\Theta^{(2)}(\mu)\right)\mathrm{d}\mu. \tag{S4}$$

Spelman and Lauga now give the non-dimensional thrust force as a sum of two terms with

$$\alpha = 2\pi\alpha_1 + \Gamma. \tag{S5}$$

The first term contains the deformation coefficient α_1, whereas the second term Γ is lengthy and neglected here to obtain a simple approximative result. This approximation is in line with the fact that a precise measurement of the surface oscillations in experiments is challenging due to limitations in temporal and spatial resolution.

Applying $P_1(\mu)=\mu$, partial integration, and a coordinate transformation with $\mu=\cos(\theta)$, α_1 can be written as

$$\alpha_1 = \frac{3}{2}\int_0^\pi \sin^2(\theta)\,\Theta^{(2)}(\theta)\,\mathrm{d}\theta. \tag{S6}$$

If we neglect Stokes drift, we obtain $\Theta^{(2)}(\theta)$ via the tangential component of the surface streaming velocity. Next, we approximate the tangential surface streaming velocity v to be constant along the bubble surface:

$$\epsilon^2\,\Theta^{(2)}(\theta) = \frac{v}{R\omega}\chi_{0\le\theta\le\theta_c}. \tag{S7}$$

Here, χ is the indicator function and θ_c is the angle corresponding to the bubble boundary. The prefactor $1/(R\omega)$ non-dimensionalizes v which can be measured using tracer particles. If R is large, then θ_c is small, so the coefficient α_1 simplifies to

$$\alpha_1 \approx \frac{3}{2}\int_0^{\theta_c}\theta^2 d\theta \frac{v}{R\omega\epsilon^2} = \frac{\theta_c^3}{2}\frac{v}{R\omega\epsilon^2}. \tag{S8}$$

Thus, the (dimensional) thrust force exerted by one bubble on the artificial muscle can be estimated as

$$F \approx \pi\,\rho\omega\theta_c^3 R^3 v \approx \pi\,\rho\omega R_c^3 v, \tag{S9}$$

where we introduced the cavity radius $R_c = R\sin(\theta_c)$ and again applied the large-R assumption. Since we expect ν to have a negative sign (see the streaming inset in **Supplementary Fig. 24**), we expect a downwards oriented force leading to a bending away from the microbubble array.

Supplementary Note 2. Deformation of the artificial muscle

We model the artificial muscle as a slender, inextensible beam divided into discrete segments corresponding to patterned microbubble arrays (**Fig. 2e**, **Supplementary Figs. 6** and **25**). The position along the beam of length L is parameterized by the coordinate $s \in [0, L]$. Due to symmetry and the nature of the applied thrust, deformation is confined to a plane and fully described by the local slope angle $\theta(s)$.

The strain energy of the slender beam due to bending is expressed as

$$V = \frac{1}{2}\int_0^L EI\theta'^2 \mathrm{d}s. \tag{S10}$$

with E as Young's modulus and I as the second moment of area. The clamped-free boundary conditions are

$$\theta(0) = 0,\ \theta'(L) = 0,\ \delta\theta(0) = 0. \tag{S11}$$

The variation in strain energy is given by:

$$\delta V = \int_0^L EI\theta'\delta\theta' \mathrm{d}s = -\int_0^L (EI\theta')'\delta\theta \mathrm{d}s. \tag{S12}$$

To evaluate the virtual work done by the external force, we compute the beam's deformation vector $(\delta x, \delta y)$:

$$\begin{pmatrix} x \\ y \end{pmatrix}(s) = \int_0^s \begin{pmatrix} \cos(\theta(s')) \\ \sin(\theta(s')) \end{pmatrix} \mathrm{d}s', \tag{S13}$$

$$\delta\begin{pmatrix} x \\ y \end{pmatrix}(s) = \int_0^s \begin{pmatrix} -\sin(\theta(s')) \\ \cos(\theta(s')) \end{pmatrix} \delta\theta(s') \mathrm{d}s'. \tag{S14}$$

Assuming a perpendicular force density $P_{ui} = n_i F_i / S_i$, where n_i, F_i, and S_i denote the number of bubbles, the thrust force of a single bubble, and the coverage area at the i-th segment, respectively, the external virtual work simplifies as

$$\begin{aligned} \delta W &= \int_0^L P_u(s) \begin{pmatrix} -\sin(\theta(s)) \\ \cos(\theta(s)) \end{pmatrix} \cdot \int_0^s \begin{pmatrix} -\sin(\theta(s')) \\ \cos(\theta(s')) \end{pmatrix} \delta\theta(s') \mathrm{d}s' \mathrm{d}s \\ &= \int_0^L \delta\theta(s) \int_s^L P_u(s') \cos(\theta(s) - \theta(s')) \mathrm{d}s' \mathrm{d}s. \end{aligned} \tag{S15}$$

Since the beam's inertia is much larger than that of the bubble, in equation (S15) the Stokes-drift-like term involving the higher-order oscillatory component of the angle θ and the lower-order oscillatory component of the force density P_u was neglected right from the beginning. That term would depend on the sound direction and vanish if its wave vector is perpendicular to the bending plane. From the principle of minimum potential energy

$$\delta V - \delta W = 0, \tag{S16}$$

and arbitrariness of $\delta\theta$, follows that

$$EI\theta'' + (EI)'\theta' + \int_s^L P_u(s')\cos\left(\theta(s) - \theta(s')\right)\mathrm{d}s' = 0, \tag{S17}$$

which is the second order integro-differential equation governing θ. Equation (S17) can be solved numerically for s going from L to 0, where the boundary condition at 0 is obeyed afterwards by adding some constant to θ. However, we want to give an analytical expression. As a first (linear) approximation of small differences in θ along the beam, one can write

$$EI\theta'' + (EI)'\theta' + \int_s^L P_u(s')\mathrm{d}s' = 0. \tag{S18}$$

To deal with the variable-size microbubble array artificial muscle, we subdivide the beam into N segments with endpoints s_j (j=1,…,N) and lengths L_j (j=1,…,N), and replace the arguments of both θ in the integrand by their nearest higher segment endpoints. For $N \to \infty$, with segment length vanishing, the approximation becomes exact. We now assume E, I, and P_u to be constant along each segment. From Eq. (S17), we get the following interface condition: $EI\theta'$ must be continuous. In each segment $s_{j-1} \le s \le s_j$ on the other hand, θ is governed by

$$E_j I_j \theta'' + (s_j - s)P_{u,j} + \sum_{i=j+1}^{N} P_{u,i} L_i \cos\left(\theta(s_i) - \theta(s_j)\right) = 0, \tag{S19}$$

where the cosine term reflects our improved approximation. This system of equations is solved for j=N up to j=1. The initial conditions for the first step are $\theta'(L)$=0 and $\theta(L)$=arbitrary. Later, continuity of $EI\theta'$ is enforced and in the end, $\theta(0)$=0 makes us add a constant on θ everywhere. θ is a third-order polynomial in s in every segment.

Applying the aforementioned algorithm for N=3, we successively get the solution for θ_i using the auxiliary variables $C_i, D_i, \varphi_i, \vartheta_i$ as follows:

$$D_2 = \frac{P_{u,3}}{2}L_3^{\,2},\ \varphi_2 = \frac{P_{u,3}}{6E_3I_3}L_3^{\,3},\ C_2 = P_{u,3}L_3\cos(\varphi_2), D_1 = \frac{P_{u,2}}{2}L_2^{\,2} + C_2L_2 + D_2,$$

$$\varphi_1 = \frac{1}{E_2I_2}\left(\frac{P_{u,2}}{6}L_2^{\,3} + \frac{C_2}{2}L_2^{\,2} + D_2L_2\right),\ C_1 = P_{u,2}L_2\cos(\varphi_1) + P_{u,3}L_3\cos(\varphi_1 + \varphi_2),$$

$$\vartheta_1(s + s_1) = \frac{1}{E_1I_1}\left(\frac{P_{u,1}s^3}{6} - \frac{C_1s^2}{2} + D_1s\right),\ \theta_1(s) = \vartheta_1(s) - \vartheta_1(0),$$

$$\vartheta_2(s + s_2) = \frac{1}{E_2I_2}\left(\frac{P_{u,2}s^3}{6} - \frac{C_2s^2}{2} + D_2s\right),\ \theta_2(s) = \vartheta_2(s) - \vartheta_2(s_1) + \theta_1(s_1),$$

$$\vartheta_3(s + s_3) = \frac{P_{u,3}s^3}{6E_3I_3}, \theta_3(s) = \vartheta_3(s) - \vartheta_3(s_2) + \theta_2(s_2). \tag{S20}$$

The resulting Y-direction deformation as a function of s in the i-th segment is then given by

$$\Delta(s) = \int_0^s \sin\left(\theta(s')\right)\mathrm{d}s'. \tag{S21}$$

Our model is also applicable to artificial muscles featuring a uniform-size microbubble array. For N=1, the slope angle at location s is

$$\theta(s) = \frac{P_u}{6EI}\left((s-L)^3 + L^3\right), \quad \text{(S22)}$$

and the deformation at the end point is

$$\Delta_y = \int_0^L \sin\left(\theta(s)\right)\mathrm{d}s. \quad \text{(S23)}$$

Supplementary Note 3. Quantitative comparison

To enable quantitative comparison with experiments, we consider the measured results of the uniform-size microbubble array artificial muscle, approximately subjected to a uniformly distributed orthogonal thrust P_u. As an example, we calculate the thrust force for a microbubble with a radius of 40 μm using **Eq. (S9)**. Based on the experimentally measured velocity of 2.5 mm/s at an excitation frequency of 27.6 kHz and input voltage of 60 V_{PP} (**Extended Data Fig. 3b**), and assuming a water density of 1000 kg/m^3, the resulting thrust force is 87 nN per bubble. Scaling this to an array of approximately 10,000 uniformly sized microbubbles on a 30 mm × 5 mm artificial muscle yields a total force of ~870 μN, corresponding to a force density of 5.8 μN/mm^2 under uniform distribution. Additional calculations for microbubbles with radii of 20 μm, 30 μm, and 40 μm across a voltage range of 1–60 V_{PP} are shown in **Supplementary Fig. 4**, with maximum force densities ranging from 5.2 to 7.6 μN/mm^2 at 60 V_{PP}.

Using Eqs. (S9), (S22), and (S23), we calculated the deformation of the artificial muscle by incorporating the experimentally measured streaming velocities of 40 μm-radius microbubbles at different voltages, and compared the results with experimental data (**Supplementary Fig. 26**). Both experimental and theoretical results reveal a monotonic increase in deformation with voltage, consistent with the expected nonlinear response under acoustic excitation. The deviation could arise from fabrication-induced imperfections and residual stresses that modify the effective stiffness.

Supplementary Note 4. Simulation setups

1. Acoustic pressure distribution in the smaller chamber

We employed a three-dimensional geometry modeled with the same dimensions as the experimental setup. A circular piezoelectric transducer (27 mm × 0.54 mm) is affixed to the glass substrate (24 mm × 60 mm × 0.18 mm). A square PDMS acoustic chamber (10 mm × 10 mm × 5 mm) is positioned in front of the transducer, which is filled with deionized water and covered with a cover glass (22 mm × 22 mm × 0.18 mm). The simulation domain included the following materials: water (built-in material), cover glass, and glass substrates (CompoGlass, built-in material), a piezoelectric transducer (PZT-5H, built-in material) (with parameters extracted from experiments). The physics modules used comprised solid mechanics, electrostatics, frequency-domain pressure acoustics, heat transfer in solids and fluids, and creeping flow. Multiphysics couplings accounted for acoustic–structure boundary interactions, acoustic streaming domain

couplings, acoustic streaming boundary couplings, and the piezoelectric effect. A user-controlled (fine) mesh was applied, and the excitation frequency was set to 33 kHz and the voltage to 60 V_{PP}.

2. Acoustic pressure distribution in the bigger chamber

We employed a three-dimensional geometry modeled with the same dimensions as the experimental setup: a thin plastic tank with dimensions of 10 cm ×10 cm × 8 cm and a thickness of 2 mm. The circular piezoelectric transducers (27 mm × 0.54 mm) are affixed to the inside surfaces and the center of the bottom surface of the tank, which is filled with deionized water. The simulation domain included the following materials: water (built-in material), a piezoelectric transducer (PZT-5H, built-in material) and a plastic chamber (polyethylene terephthalate). The physics modules used comprised solid mechanics, electrostatics, frequency-domain pressure acoustics, heat transfer in solids and fluids, and creeping flow. Multiphysics couplings accounted for acoustic–structure boundary interactions, acoustic streaming domain couplings, acoustic streaming boundary couplings, and the piezoelectric effect. A user-controlled (fine) mesh was applied, and the excitation frequency was set to 33 kHz and the voltage to 60 V_{PP}.

3. Acoustic streaming

To address the computational complexity and time constraints of 3D streaming flow simulations, we employed a simplified 2D geometric model. This model features three arch geometries representing bubbles of different radii arranged sequentially, each size comprising five microbubbles. The displaced region shown in **Extended Data Fig. 2d** represents a zoomed-in view of an acoustic chamber embedded within a 10 mm × 10 mm water domain. The air bubbles and surrounding fluid were modeled using Thermoviscous Acoustics, coupled to a Pressure Acoustics domain with a Spherical Wave Radiation boundary condition. Surface tension effects at the bubble interface were incorporated via the Surface Tension boundary condition. A rectangular piezoelectric transducer (PZT-5H material) was affixed to the chamber base. The simulation domain included water (built-in material) and the PZT-5H transducer. For flow tracking, 1 µm-radius particles were uniformly distributed in the chamber. Key multiphysics couplings include acoustic–structure boundary interactions, acoustic streaming (domain and boundary couplings), and piezoelectric effects. A user-controlled fine mesh was applied, and excitation frequencies of 30 kHz, 80 kHz, and 100 kHz were tested.

4. Oscillation shape of the variable-size beam

We employed a three-dimensional model of an artificial muscle, representing a PDMS structure with dimensions of 3 cm × 0.5 cm × 0.1 cm, simulated using the Solid Mechanics module. The micro-cavities within the muscle were simplified into three distinct arrays with a reduced number of cavities to streamline the computational model. In this simulation, the central region of the muscle was excited by applying a uniform stress (force per unit area) to each cavity, and the time-dependent solution was recorded at 0.5 s. The boundary conditions were set to clamped-free, with the fixed edges constrained, while the free edges were allowed to deform. The PDMS material properties were defined with a Young's modulus of 1 MPa and a Poisson's ratio of 0.1.

Supplementary Note 5. Compactness and weight characteristics of the artificial muscle

The compactness and weight characteristics of the artificial muscle are:

1. A high compactness of 3,000 microbubbles/mm²

The smallest microbubble used in the experiments has a diameter of 12 μm and a spacing equal to the radius of each bubble. Thus, the total number of bubbles within 1 mm^2 is approximately $(1000\ \mu m/(3\times 6\ \mu m))^2 \sim 3000$.

2. A low weight of 0.047 mg/mm²

For an artificial muscle with dimensions of 5 mm × 3 mm and a thickness of 70 μm, the measured weight is 0.7 mg, corresponding to a weight per unit area of 0.047 mg/mm^2.

Supplementary Figures

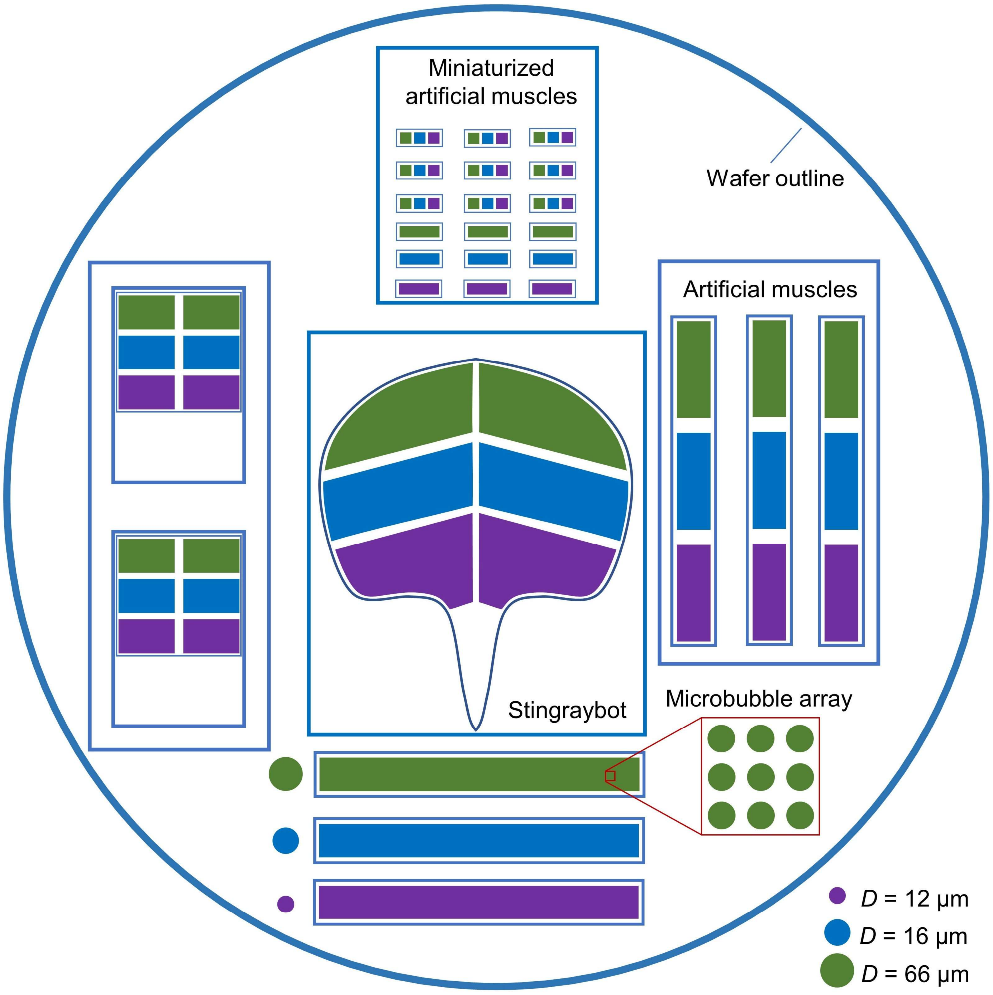

Supplementary Fig. 1 | Design of artificial muscles and stingraybot. At the center of the 4-inch wafer profile, a bioinspired stingraybot is depicted, comprising three distinct microbubble arrays with varying radii arranged from the head to the tail. At the bottom are three artificial muscles with uniform-size microbubble arrays. On the right are the artificial muscles with variable-size microbubble arrays. Both the artificial muscles at the bottom and the right side are 30 mm in length and 5 mm in width. The top panel shows the miniaturized artificial muscles, scaled down by a factor of 0.12 compared to those at the bottom and on the right. Despite the size reduction, the microbubbles in the miniaturized muscles have the same radii. The left panel shows paddles containing variable-size microbubble arrays with identical bubble radii, designed to generate turning forces for navigation.

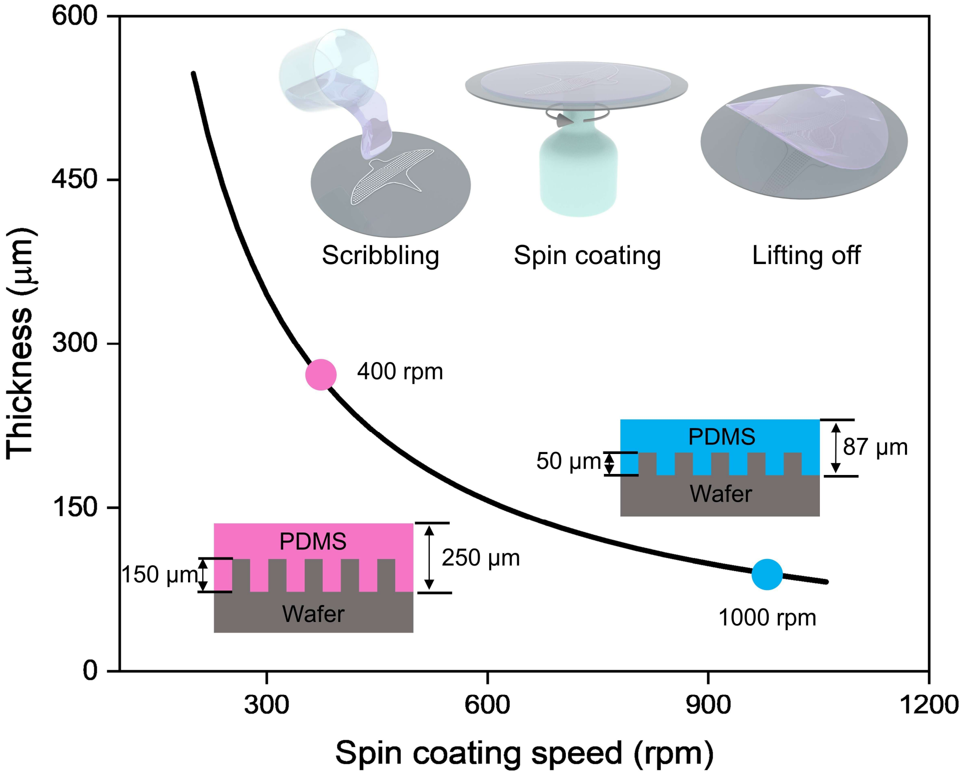

Supplementary Fig. 2 | Artificial muscle thickness versus PDMS spin coating speed. The black curve illustrates that the resulting thickness of the artificial muscle decreases with a higher spinning speed. The spinning speeds utilized in experiments are highlighted by two specific data points: The pink dot corresponds to a spinning speed of 400 rpm, which was employed on a wafer with 150 μm pillars, resulting in a larger thickness of 250 μm for the artificial muscle. The blue dot represents a spinning speed of 1000 rpm, which was applied to a wafer with 50 μm pillars, yielding a thinner artificial muscle with a thickness of 87 μm. Insets demonstrate the fabrication process.

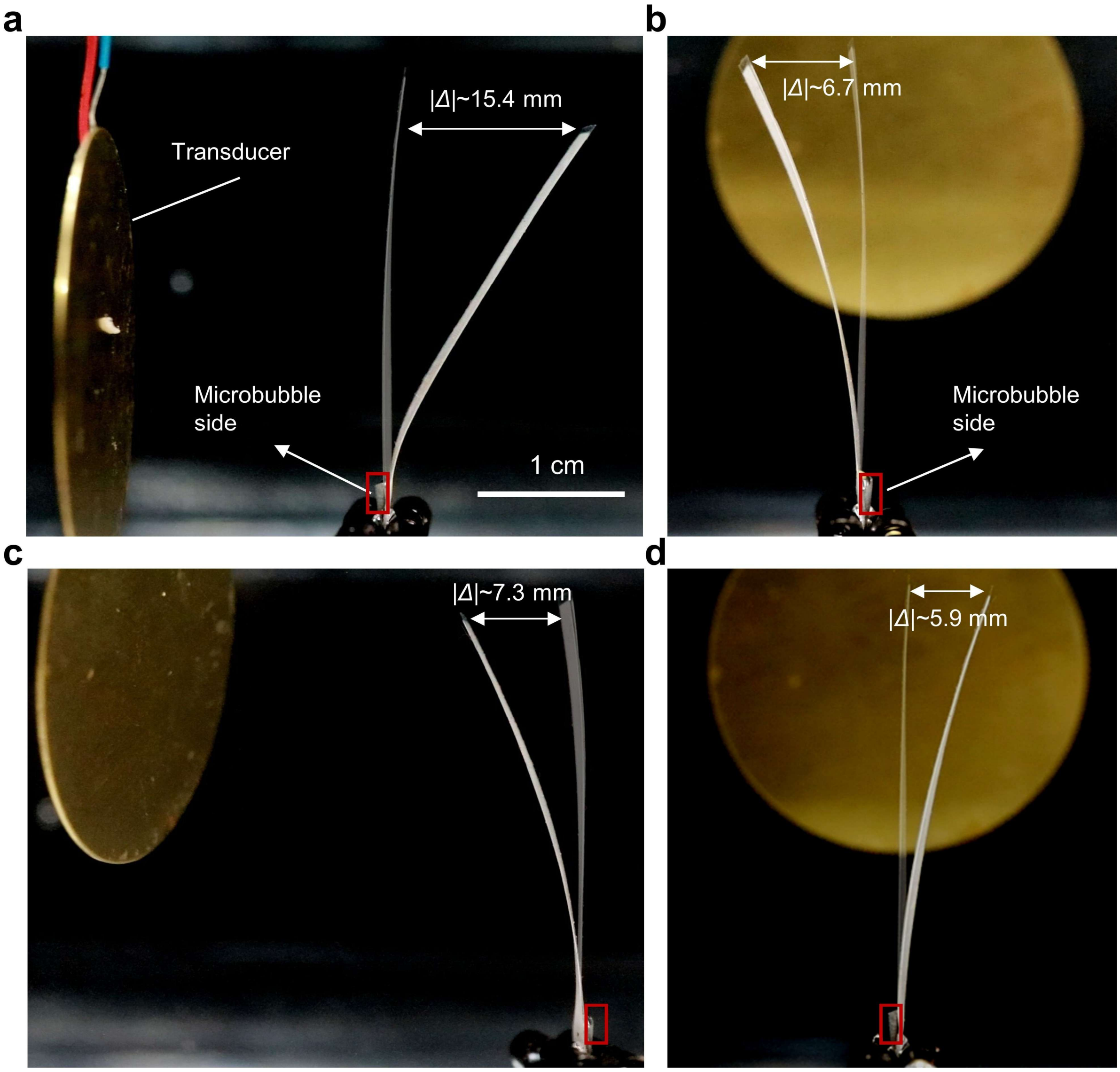

Supplementary Fig. 3 | Deformations of a uniform-size microbubble array artificial muscle with various transducer positions. a to **d,** The transducer was positioned with four distinct orientations: **a**, directly facing the microbubble-embedded side, **c**, opposite to it, and perpendicular to the arrays on the left (**b**) and right (**d**) sides of the artificial muscle. The side with the microbubble array is marked by a red rectangle. The muscle demonstrated a consistent bending direction regardless of these positions, i.e., all samples bent in the direction opposite to the microbubble-array side, despite the variations in bending amplitudes. The ultrasound excitation was 80.5 kHz and 60 V_{PP}. In **b** and **d** the bending is due to the acoustic streaming thrust. One can expect the sound wave in **a** and **c** to push the artificial muscle to the right, but the unaltered deflection in **c** indicates that a force directly due to sound reflection plays only a minor role compared to the force due to acoustic streaming.

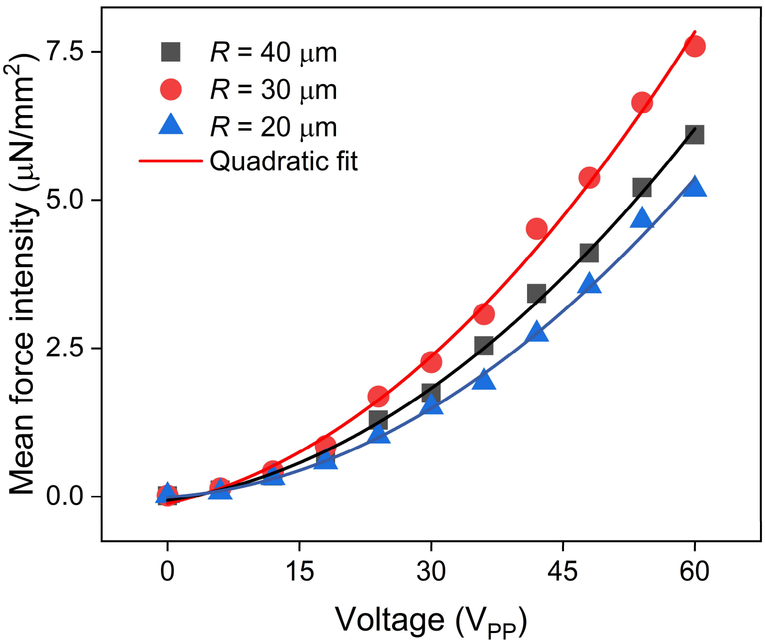

Supplementary Fig. 4 | Calculated force intensities. Calculated mean force intensities as a function of applied voltage. The dot values were obtained using Eq. (S9), incorporating experimental data from **Extended Data Fig. 3b**, and the solid lines represent quadratic fits.

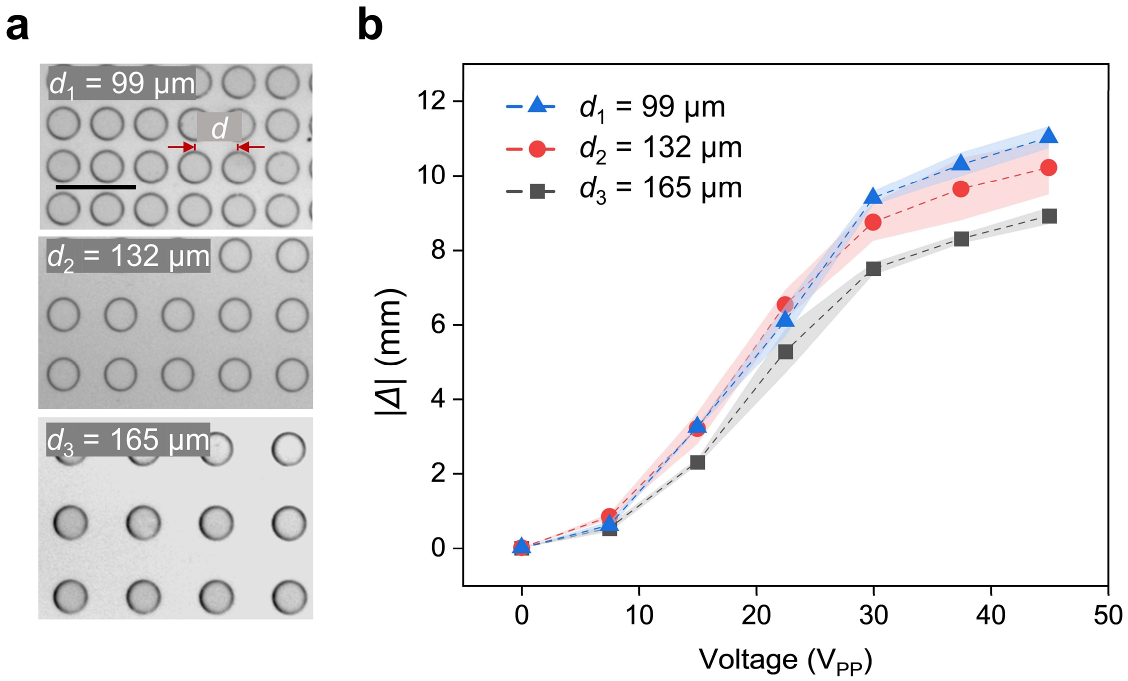

Supplementary Fig. 5 | Deformation (|Δ|) of a uniform-size microbubble array artificial muscle with varying bubble spacings. **a**, Microscopic image of the microbubble arrays (66 µm × 50 µm cavities) with different spacings (d = 99 µm, 132 µm, 165 µm), where the scale bar is 200 µm. n = 5 independent samples. **b**, Deformation of the artificial muscles with different spacings between the microbubbles as a function of the applied voltage, where the input frequency is 33.2 kHz. The shaded error bands represent mean ± s.d. from n = 5 independent measurements.

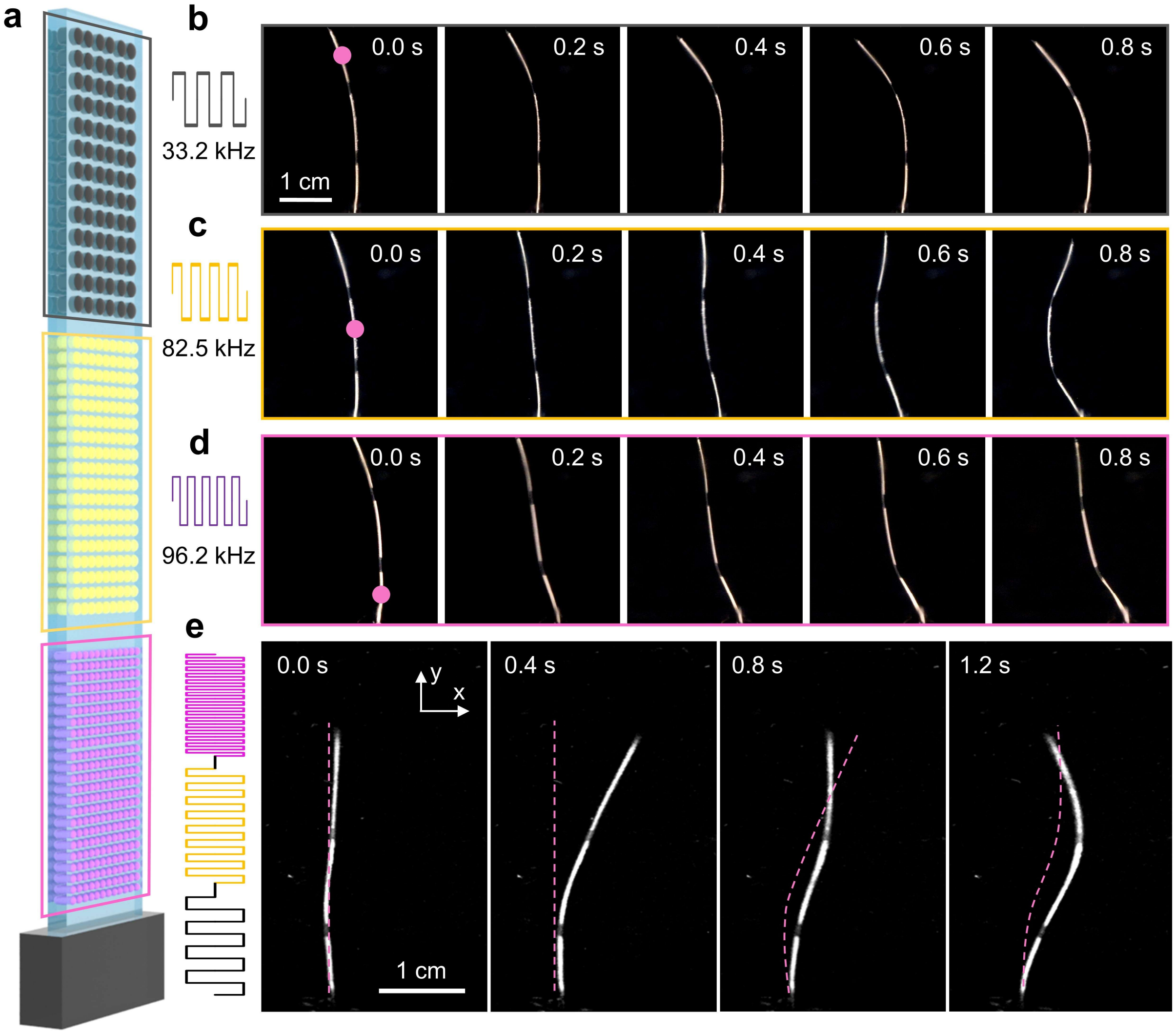

Supplementary Fig. 6 | Deformation of a variable-size microbubble array artificial muscle under continuous and sweeping-frequency ultrasound excitation. a, Schematic of the artificial muscle (thickness: 200 μm), comprising three different microbubble arrays, denoted by black (66 μm × 50 μm cavities), yellow (16 μm × 50 μm cavities), and pink (12 μm × 50 μm cavities) colors. **b** to **d,** Time-lapse sequences of the artificial muscle's deformation when we respectively excited the top, middle, and bottom microbubble arrays, each corresponding to a specific frequency indicated by a color-coded square signal. The magenta dot denotes the excited microbubble array. **e**, A time-lapse sequence image of the artificial muscle's response to sweeping frequency ultrasound excitation, ranging from 20 kHz to 90 kHz, over a sweep period of 2 seconds. The magenta dotted line denotes the posture of the artificial muscle at the previous time step. The ultrasound excitation voltage for all panels is 60 V_{PP}.

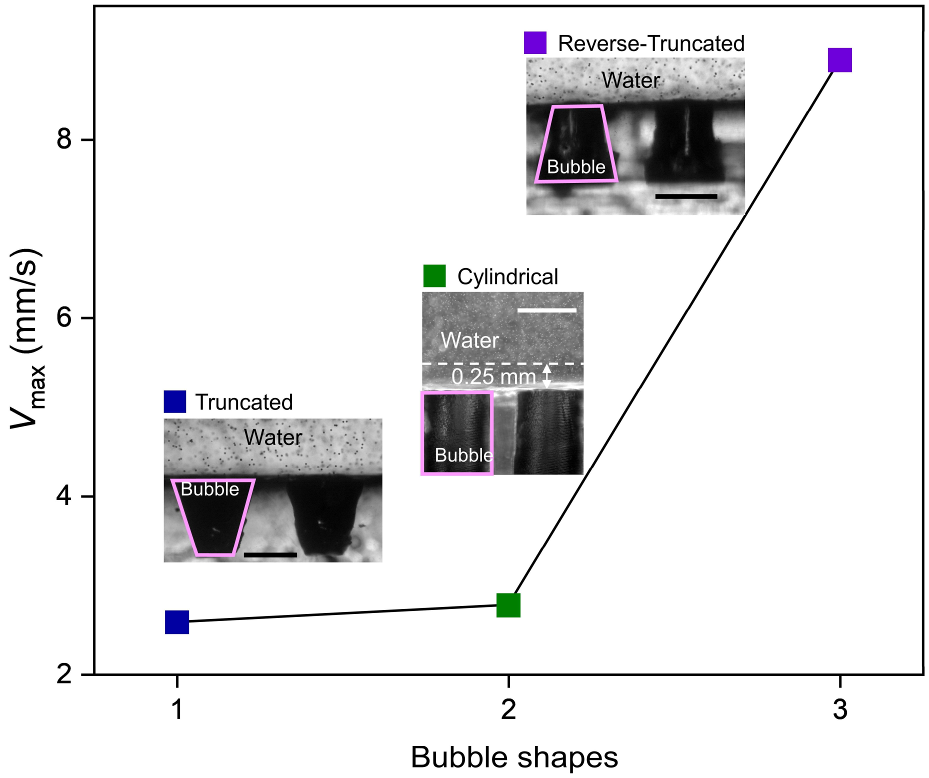

Supplementary Fig. 7 | Measured maximum streaming for three different bubble shapes. The blue, green, and purple points denote truncated, cylindrical, and reverse-truncated bubble shapes, respectively, with the streaming velocity measured along a line 0.25 mm from the bubble surface using 2 μm tracer microparticles at 2.1 kHz and 30 V_{PP} in all experiments. Scale bar: 1 mm. $n = 5$ independent samples.

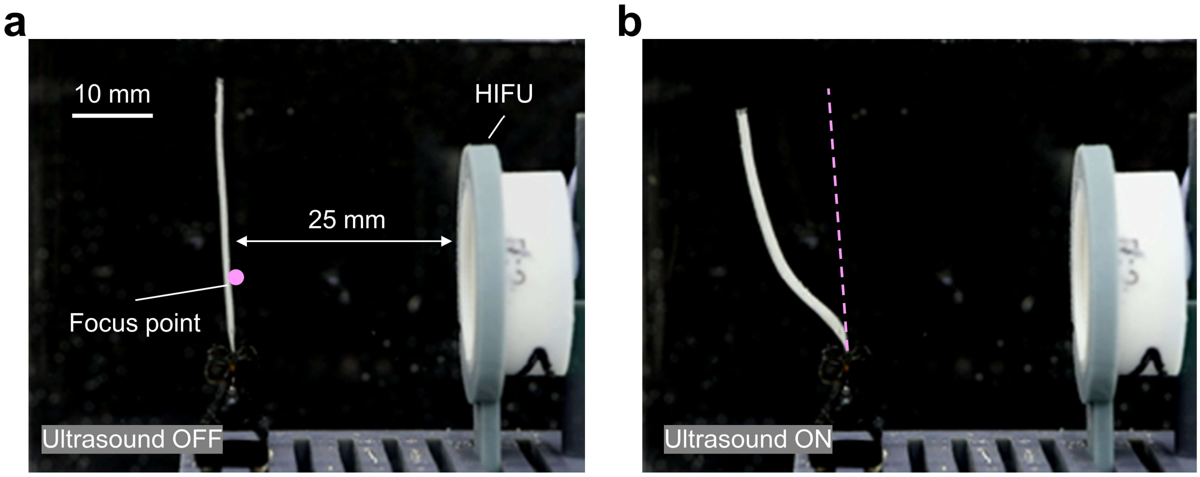

Supplementary Fig. 8 | Deformation of a uniform-size microbubble array artificial muscle with confocal ultrasound excitation. **a** and **b**, Artificial muscle before and after ultrasound excitation, respectively. The pink dashed line in **b** indicates the original position of the muscle prior to ultrasound excitation. The confocal ultrasound excitation was generated using a HIFU transducer with a focal distance of 25 mm, driven at 70 kHz and 45 V_{PP}.

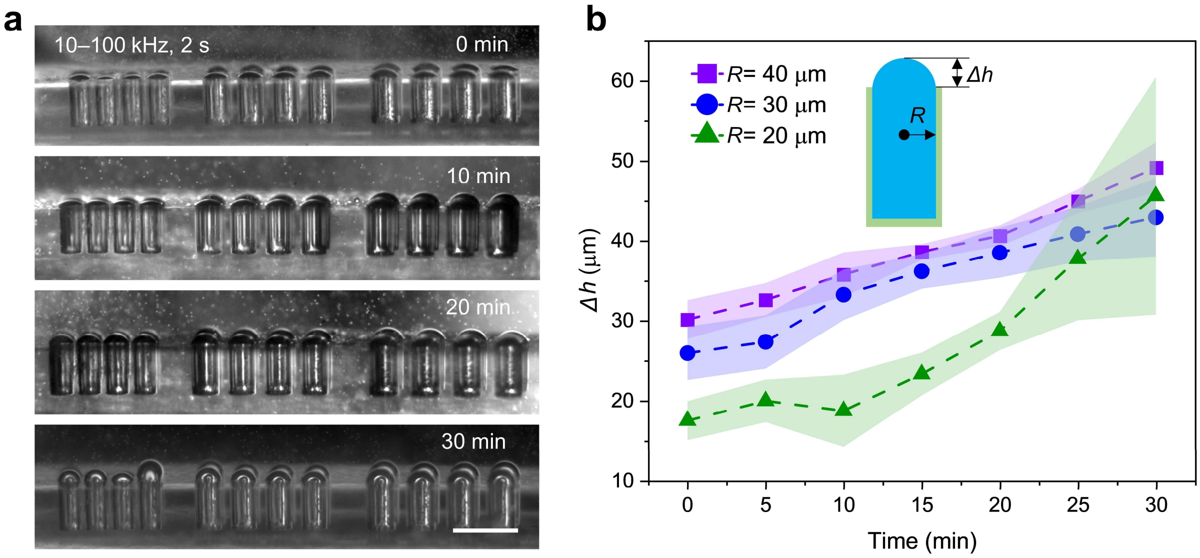

Supplementary Fig. 9 | Robustness analysis of the microbubbles at different time scales under sweeping-frequency ultrasound excitation. a, Microscopic images of the variable-size microbubble arrays at different excitation times, with an excitation frequency ranging from 10 to 100 kHz, a sweeping duty cycle of 2 s, and voltage of 60 V_{PP}. Scale bar: 200 μm. $n = 5$ independent samples. **b**, Measurement of the mean outer height change (Δh) outside the cavity for bubbles of different sizes as a function of time. The shaded error bands represent mean ± s.d. from $n = 5$ independent measurements.

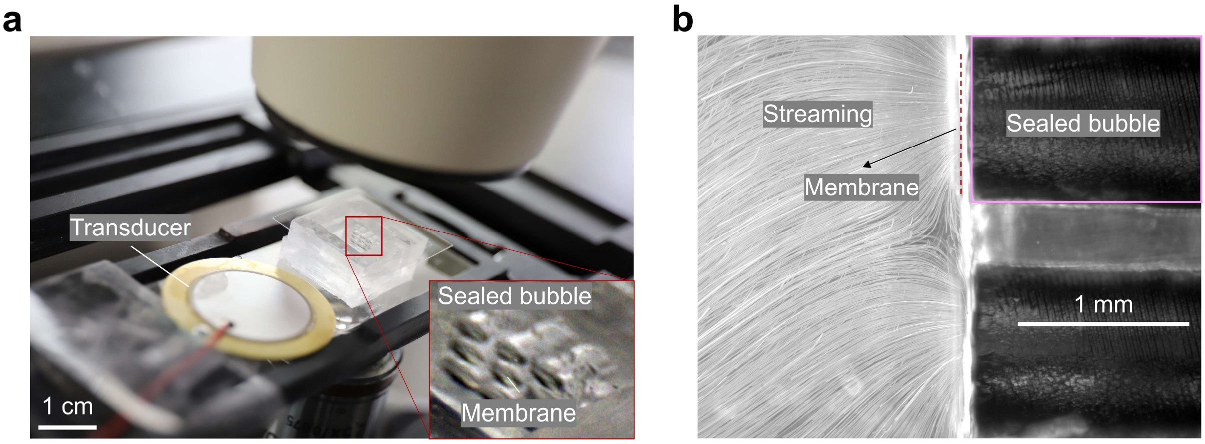

Supplementary Fig. 10 | Experimental setup and streaming of membrane-sealed bubbles. a, Experimental setup showing the transducer and a membrane-sealed 3 × 3 bubble array (1 mm diameter, 1.5 mm depth) mounted on a glass slide. The inset highlights the sealed bubbles. The membrane is 10 μm thick. **b**, Leftward microstreaming jetting generated from a microbubble array oscillating under ultrasound excitation demonstrated by 6 μm tracer microparticles. Excitation was applied at 2.1 kHz with a voltage of 60 V_{PP}. The pink rectangle represents the contour of the cross section of the sealed bubble, the red dashed line indicates the location of the membrane.

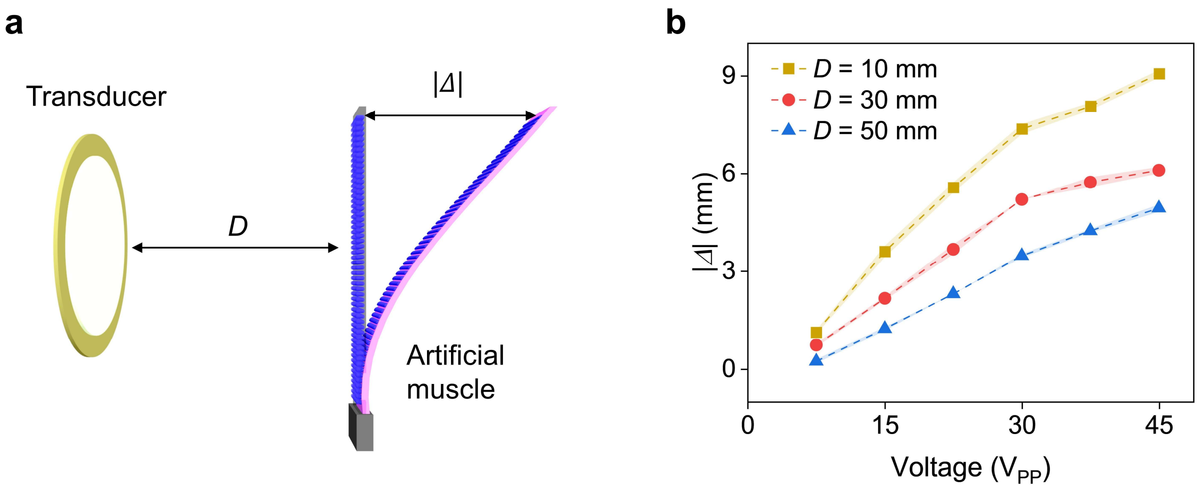

Supplementary Fig. 11 | Deformation (|*Δ*|) of a uniform-size microbubble array artificial muscle under different excitation distances (*D*). a, Schematic shows a transducer exciting a uniform-size microbubble array artificial muscle (12 μm × 50 μm cavities) at varying distances, under a fixed excitation frequency of 96.2 kHz. **b,** Measured deformation as a function of voltages at varying excitation distances. The shaded error bands represent mean ± s.d. from n = 5 independent measurements.

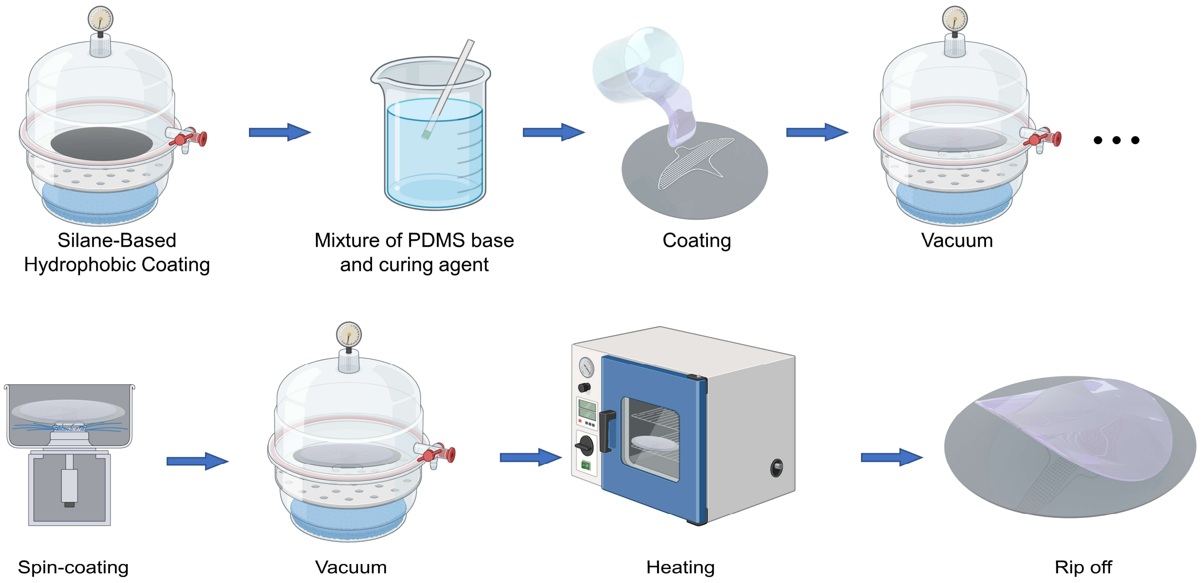

Supplementary Fig. 12 | Fabrication process of the soft artificial muscle. A 4-inch micropillar-patterned wafer was treated with a silane-based hydrophobic coating to enhance surface properties. A PDMS mixture, prepared with a 10:1 base-to-curing-agent ratio, was poured onto the wafer and degassed under vacuum (<1 mbar). Spin-coating was performed at various rotation speeds to get various thicknesses, respectively. After a second vacuum treatment, the PDMS was cured in a sequential heating process (1 hour at 60 °C, 1 hour at 80 °C, and 1 hour at 100 °C). The cured PDMS membrane was then peeled off, yielding a uniform soft layer suitable for artificial muscle and soft robotic applications. Created in BioRender. Shi, Z. (2025) https://BioRender.com/r9nsrs6.

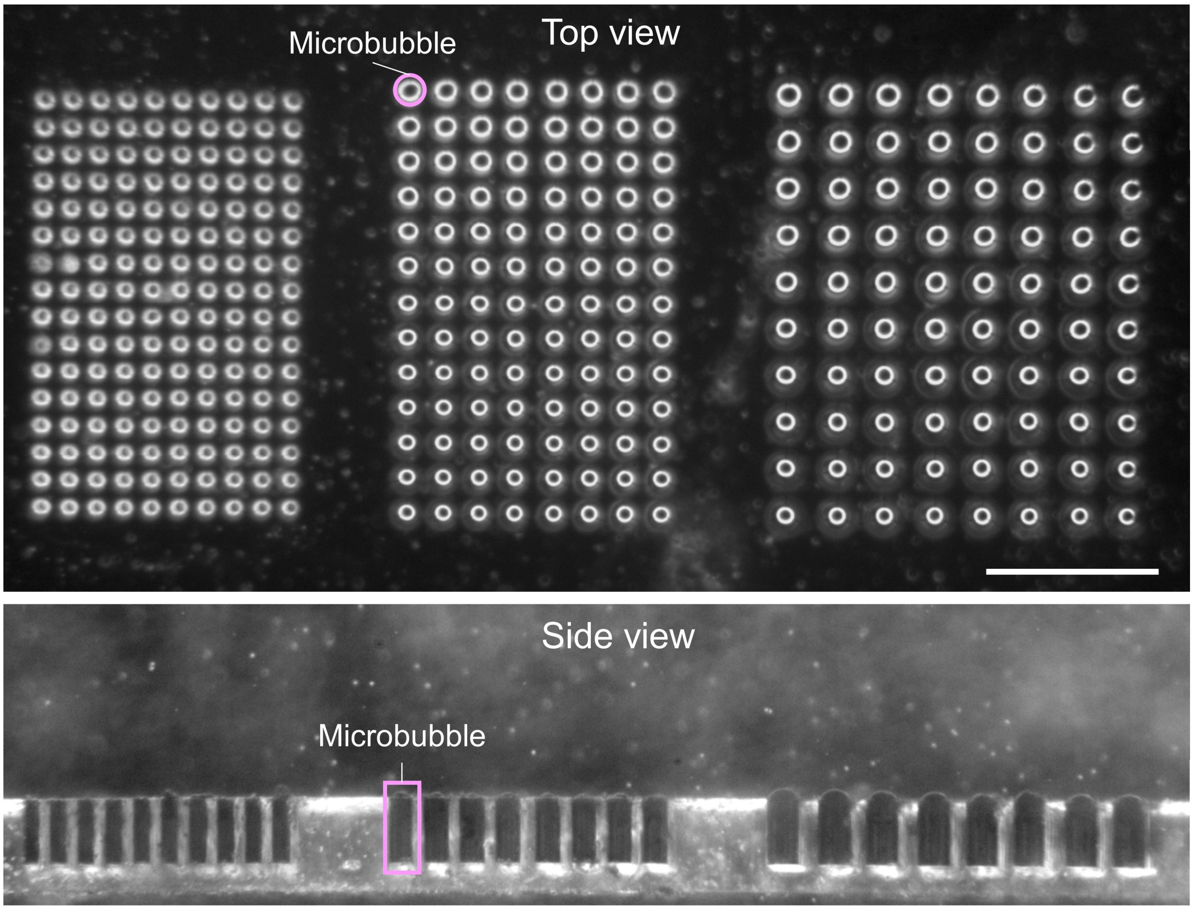

Supplementary Fig. 13 | Microscopic top and side views of microbubble arrays. The images illustrate the structure and arrangement of microbubbles. Each cavity trapped only a single bubble. From left to right, the diameters of the microbubble arrays are 20 µm, 30 µm, and 40 µm, respectively. A trapped microbubble within a cavity is marked with a pink circle (top) and a rectangle (bottom). Scale bar: 200 µm. $n = 5$ independent samples.

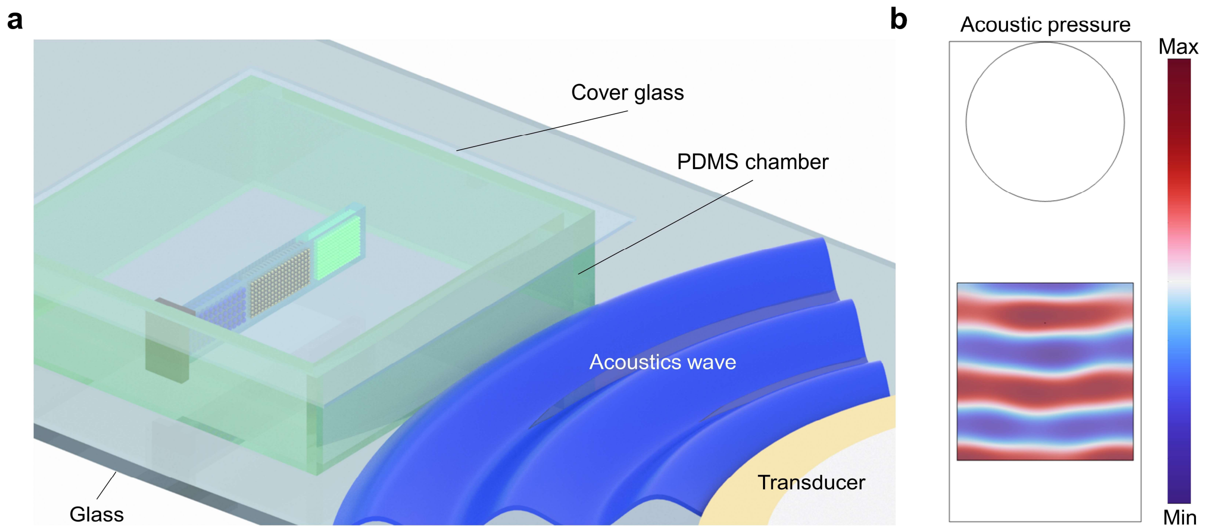

Supplementary Fig. 14 | Experimental setup for microscopic characterization of microbubble arrays and corresponding acoustic pressure field simulation. a, Experimental setup. A transducer is affixed to the glass slide to generate sound waves and a PDMS chamber is placed in front of the transducer. The artificial muscle was suspended in the center of the chamber with one end clamped to the sidewall and the other end left free. The chamber is filled with deionized water and sealed with a cover glass. **b**, Simulation of the acoustic pressure distribution in the chamber.

a

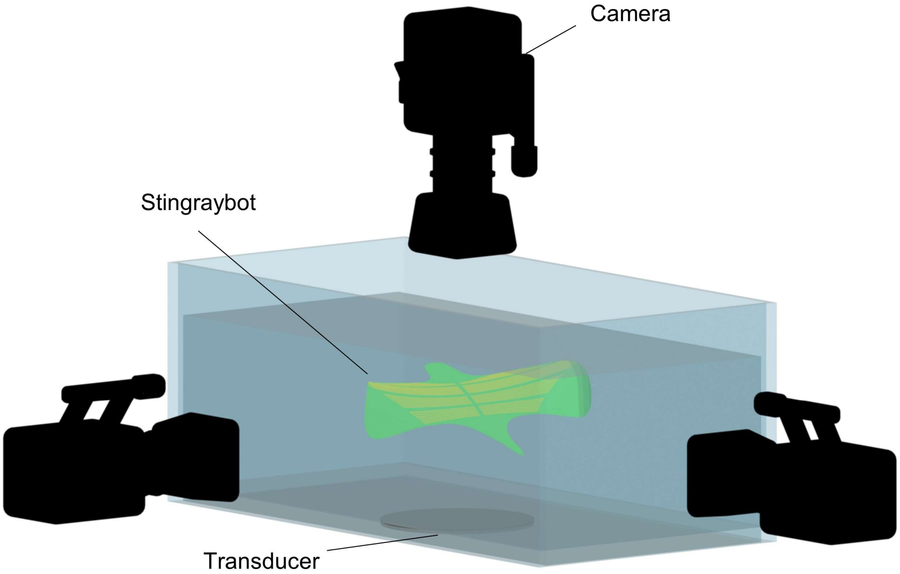

b

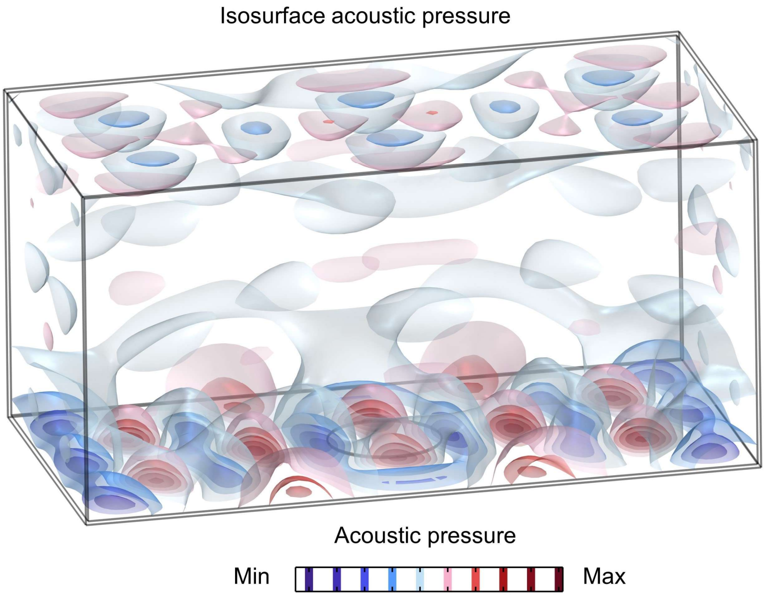

Supplementary Fig. 15 | Experimental setup for artificial muscle actuation and corresponding acoustic pressure field simulation. **a**, Experimental setup. The acoustic tank is filled with deionized water. Three cameras are positioned at the top, left, and front to capture the motion of the bioinspired stingraybot. Transducers are affixed to the bottom of the tank's wall. **b**, Simulation of acoustic pressure isosurfaces within the tank, where the pressure demonstrates no obvious pressure nodes inside the tank.

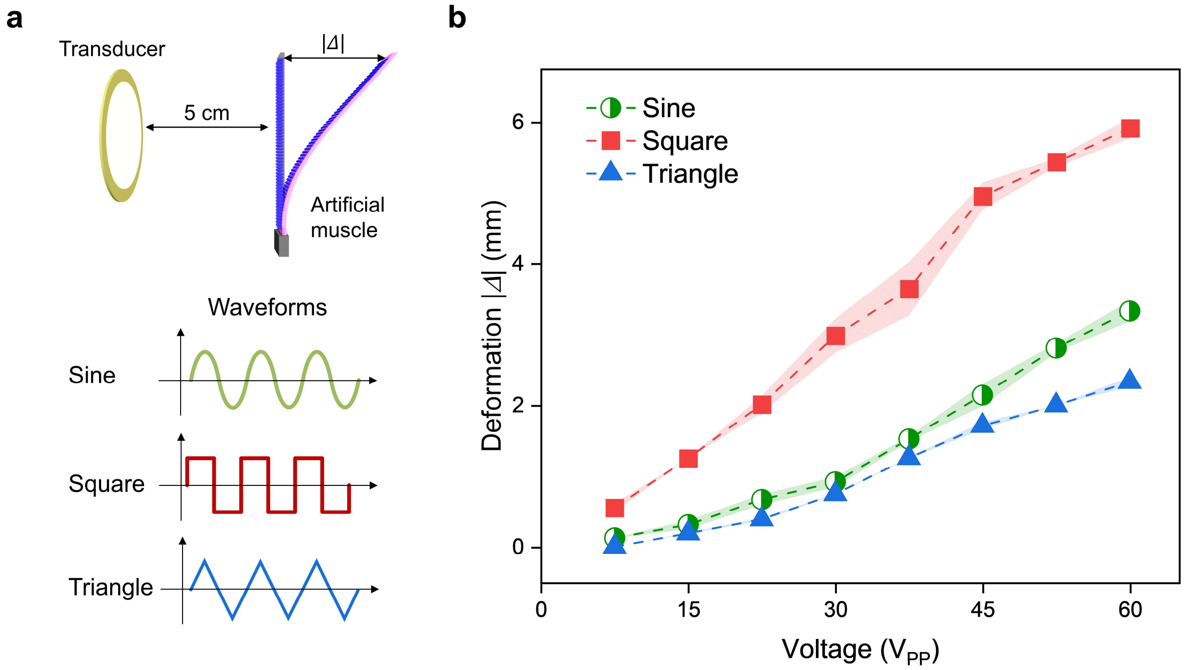

Supplementary Fig. 16 | Deformation of the artificial muscle under different waveform inputs as a function of applied voltage. **a**, Experimental setup and the waveforms of the excitation signal. The distances between the transducer and the uniform-size microbubble array (12 µm × 50 µm cavities) artificial muscle is 5 cm, the excitation frequency is set to 95.5 kHz. **b**, The deformation response for three waveform types. Shaded areas represent the standard deviation of deformation for each waveform type, indicating variability across measurements. The shaded error bands represent mean ± s.d. from n = 5 independent measurements.

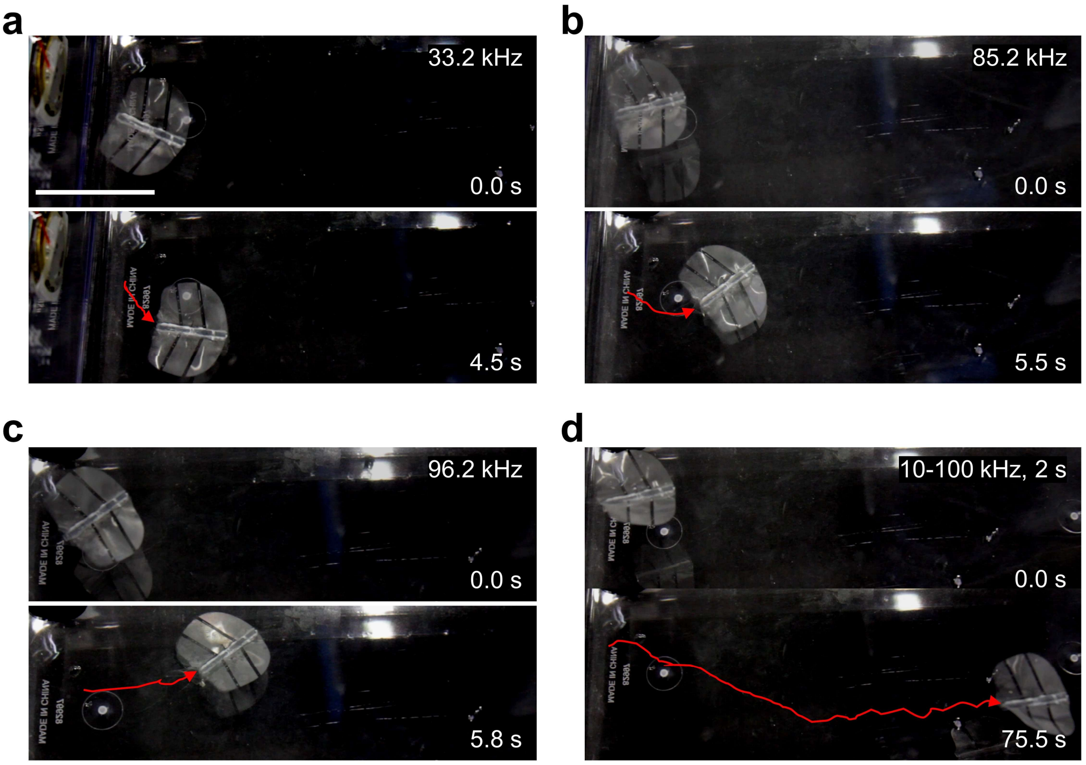

Supplementary Fig. 17 | Swimming distances of a stingraybot under different excitation conditions. **a–c**, Stingraybots with microbubble diameters of 12 μm, 16 μm, and 66 μm swim under constant-frequency excitation at 33.2 kHz, 85.2 kHz, and 96.2 kHz, respectively. **d**, Swimming of the stingraybot under frequency-sweep excitation (10–100 kHz, 2-s sweep period). All excitations are driven at 60 V_{PP}. Scale bar: 5 cm.

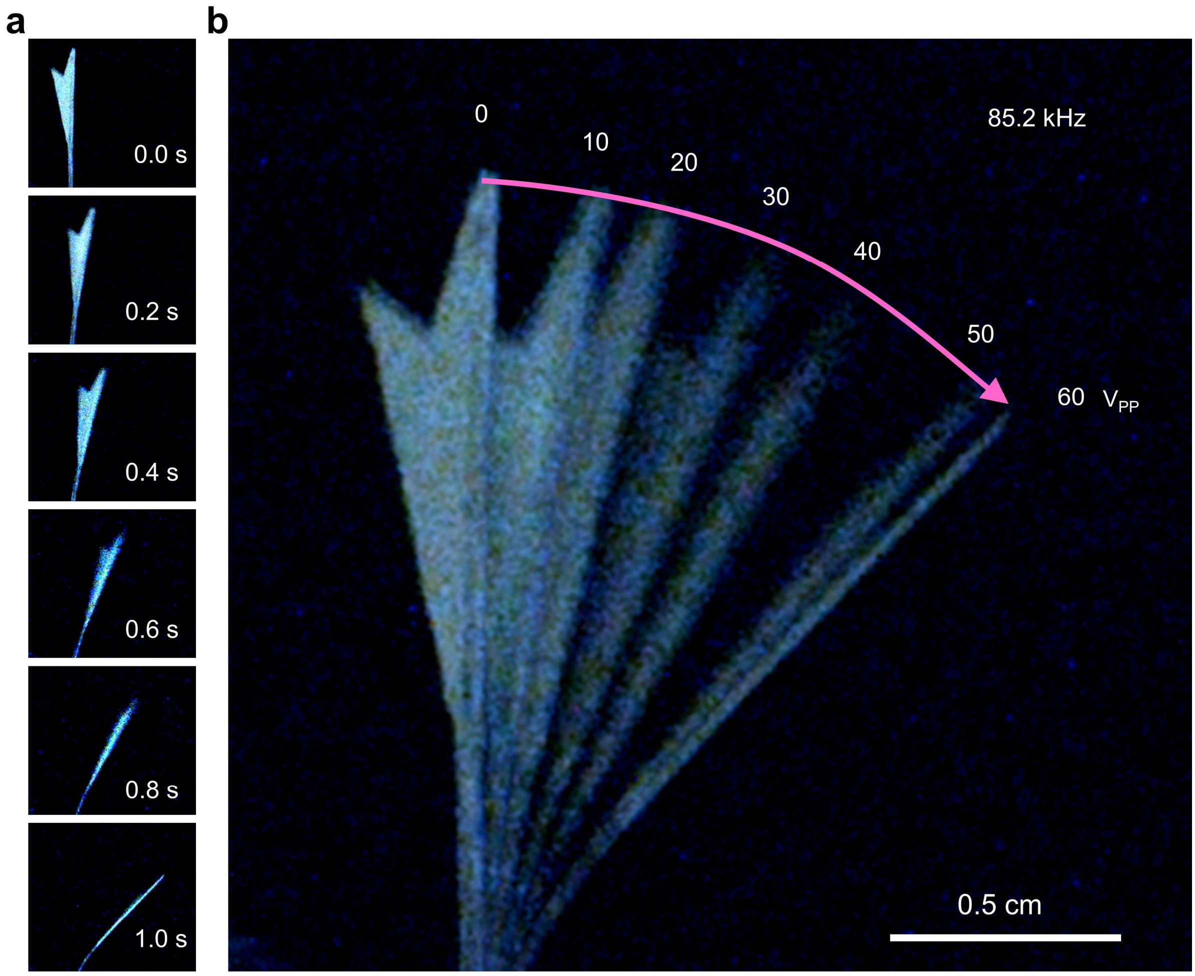

Supplementary Fig. 18 | Deformation of a robotic shark tail with the uniform-size microbubble array artificial muscle. a, Time-lapse deformation of the shark tail over a 1 s time interval at ultrasound excitation of 85.2 kHz and 60 V_{PP} with uniform-size microbubble array (16 μm × 50 μm cavities). **b,** Superimposed deformation of the shark tail with voltage increased from 0 to 60 V_{PP}, while maintaining a constant frequency of 85.2 kHz. The magenta arrow denotes the deformation direction with the microbubble array on the left side of the shark tail. n = 5 independent samples.

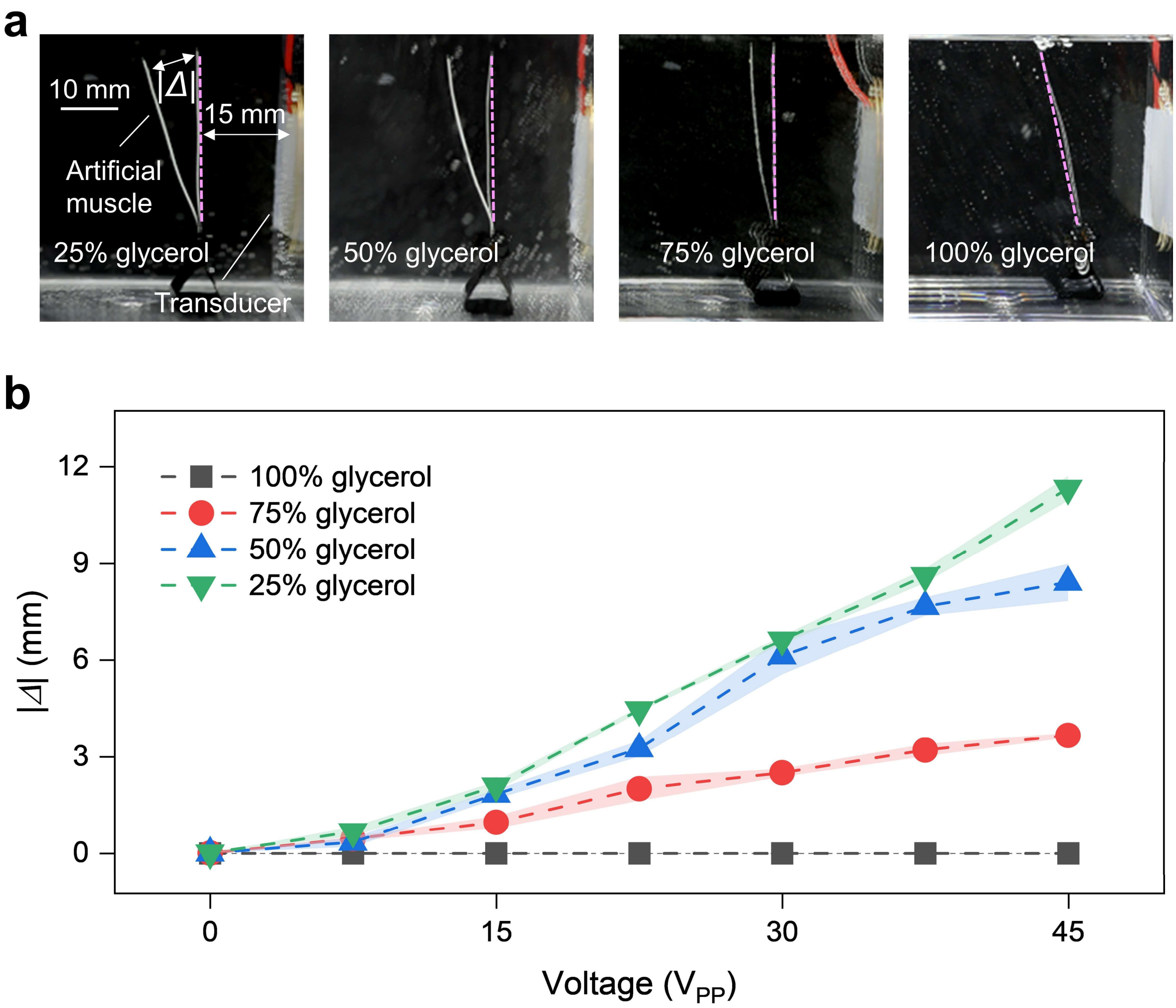

Supplementary Fig. 19 | Deformation of an artificial muscle in glycerol-water mixtures with different mixing ratios. a, Experimental results for a uniform-size microbubble array artificial muscle (12 µm × 50 µm cavities) under varying glycerol-water ratios. The distance between the transducer and the artificial muscle is 15 mm. Pink dashed lines indicate the initial position of the artificial muscle. Excitation was applied at 96.2 kHz with a voltage of 45 V_{PP}. **b**, Deformation ($|\Delta|$) of the artificial muscle as a function of the excitation voltage for different glycerol-water ratios. The shaded error bands represent mean ± s.d. from n = 5 independent measurements.

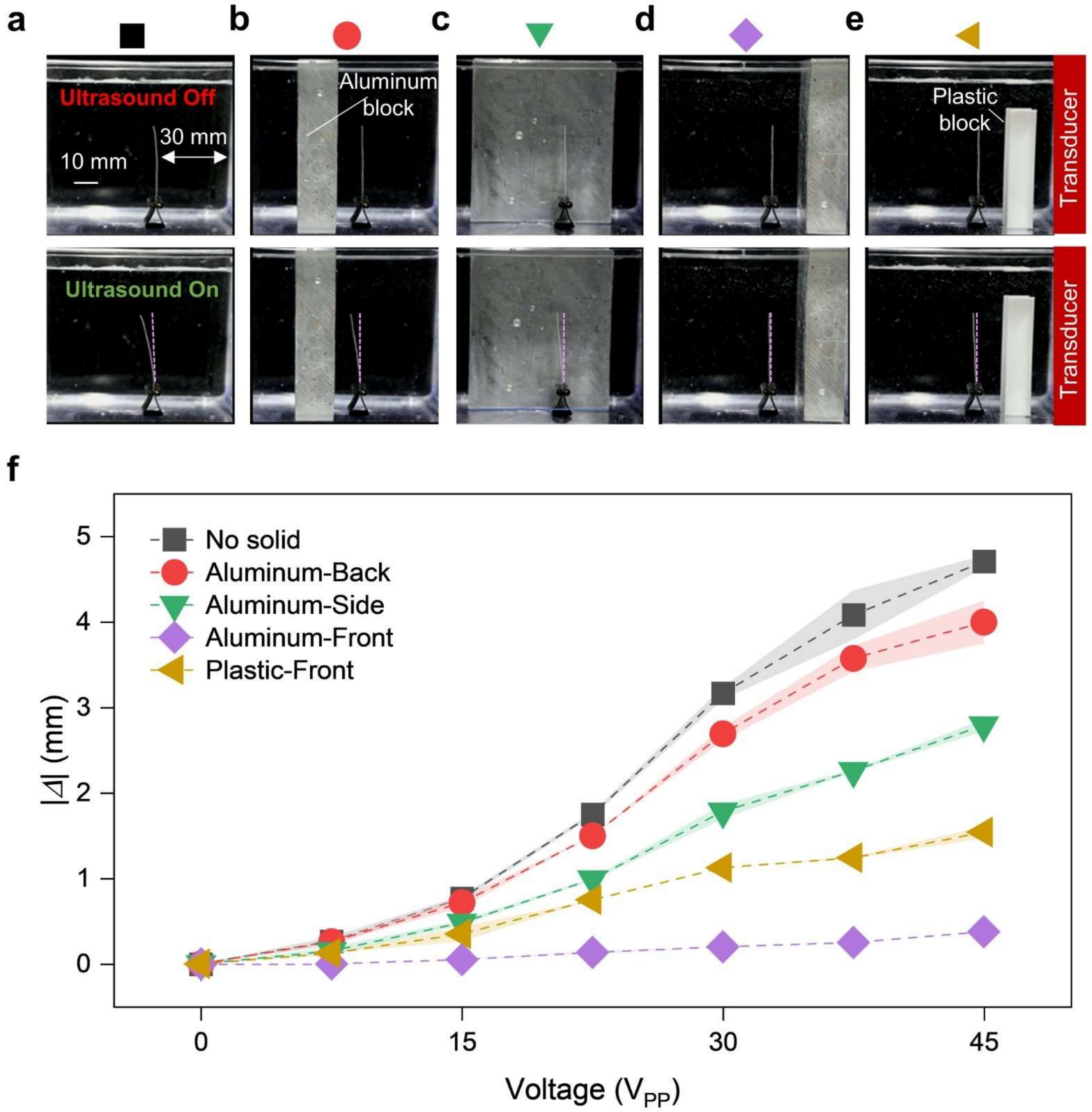

Supplementary Fig. 20 | Effect of surrounding solid media on artificial muscle deformation under ultrasound excitation. a-e, Experimental setup showing initial (pink dashed lines) and deformed positions of a uniform-size microbubble array artificial muscle (12 μm × 50 μm cavities) with various solid-media configurations: no solid (black square), aluminum at the back (red circle), side (green triangle), and front (purple diamond), and plastic at the front (yellow triangle). All solids are placed 10 mm from the muscle, with the transducer 30 mm away. Excitation was applied at 95.5 kHz with a voltage of 45 V_{PP}. **f**, Deformation ($|\Delta|$) of the artificial muscle as a function of the excitation voltage for different solid media types and positions. The shaded error bands represent mean ± s.d. from n = 5 independent measurements.

a, b

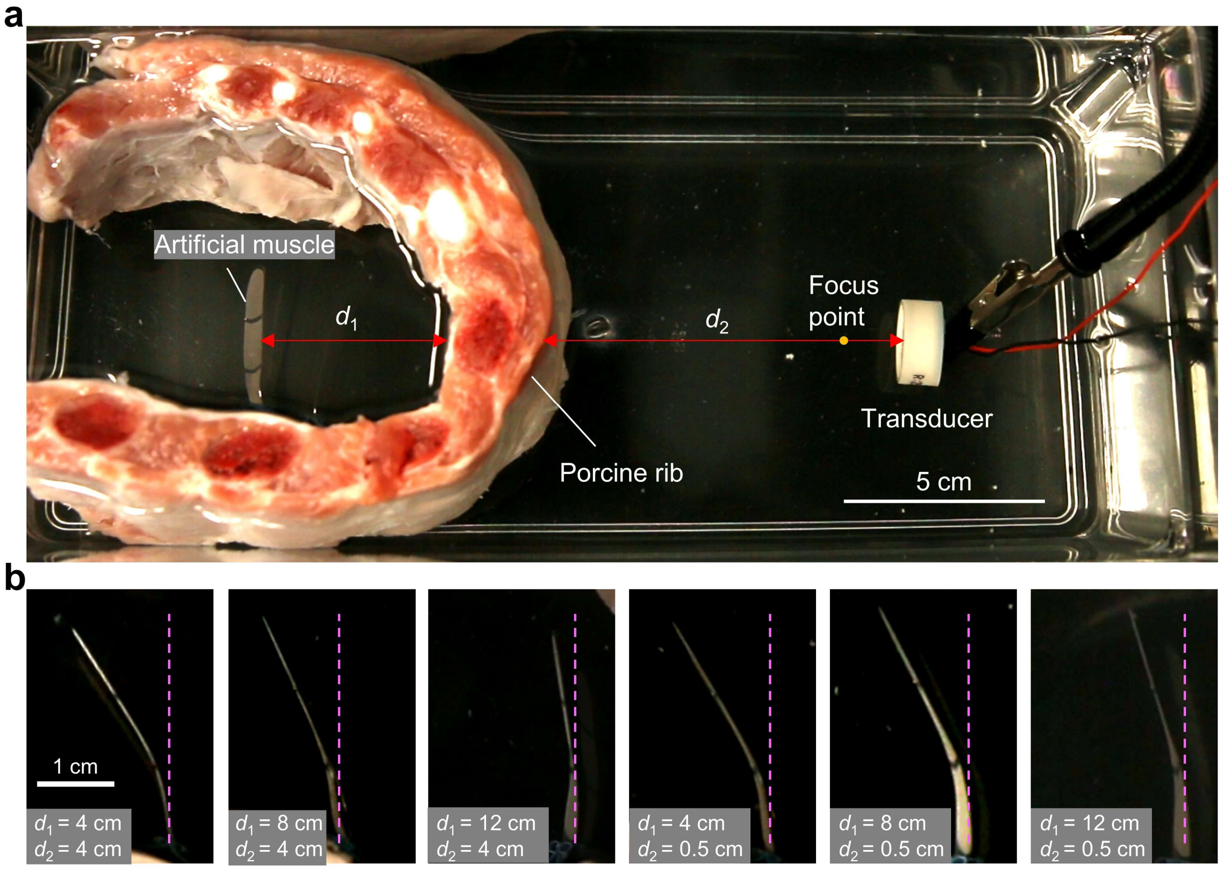

Supplementary Fig. 21 | Actuation of the artificial muscle behind porcine ribs. a, Experimental setup showing the artificial muscle positioned at distance d_1 behind a porcine rib segment, with a HIFU transducer placed at distance d_2 on the opposite side. The transducer's focal point is indicated (2 cm), but actuation was performed in the far field. **b**, Deformation of the artificial muscle under ultrasound excitation (2 MHz, 60 V_{PP}) at different positions. Left to right: d_1 = 4, 8, 12 cm (d_2 = 4 cm); d_1 = 4, 8, 12 cm (d_2 = 0.5 cm). The actuator exhibits clear deformation even through bone, demonstrating reliable performance across clinically relevant distances. Pink dashed lines indicate the initial position of the artificial muscle.

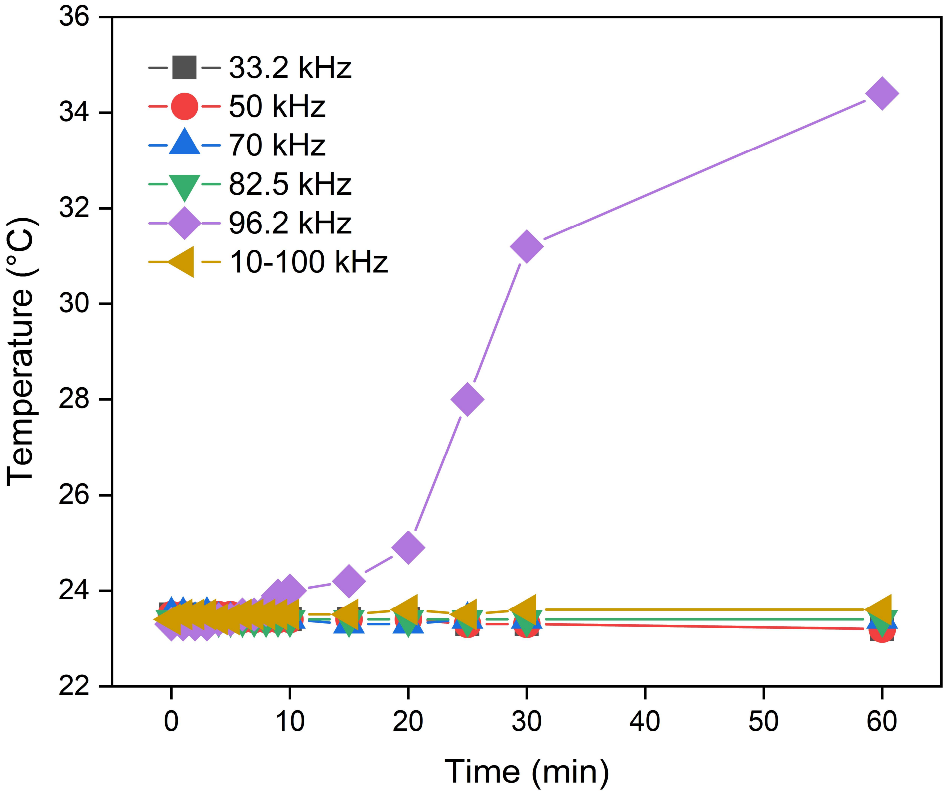

Supplementary Fig. 22 | Temperature variation for different ultrasound frequencies versus time. The temperature remains relatively stable for 33.2 kHz, 50 kHz, 70 kHz, 82.5 kHz, and 10-100 kHz with 2 seconds sweep period, whereas for 96.2 kHz there is a significant increase in temperature as time progresses. The temperature changes were measured using a temperature probe positioned ~1 mm away from the piezo transducer.

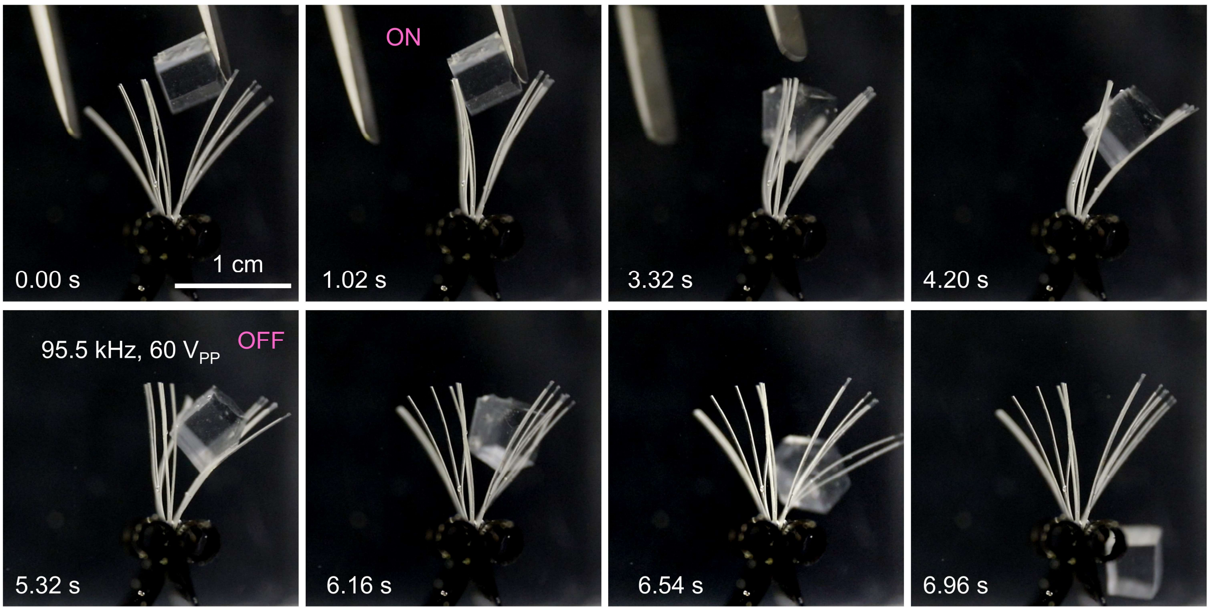

Supplementary Fig. 23 | A microbubble-array soft gripper capturing a 35 times heavier PDMS block. The gripper is 7 mg and the PDMS block is 250 mg. The soft gripper consists of 10 uniform-sized microbubble-array artificial muscles, each of them housed >20000 microbubbles with the diameter of 12 µm. The ultrasound excitation frequency and voltage are 95.5 kHz and 60 V_{PP}.

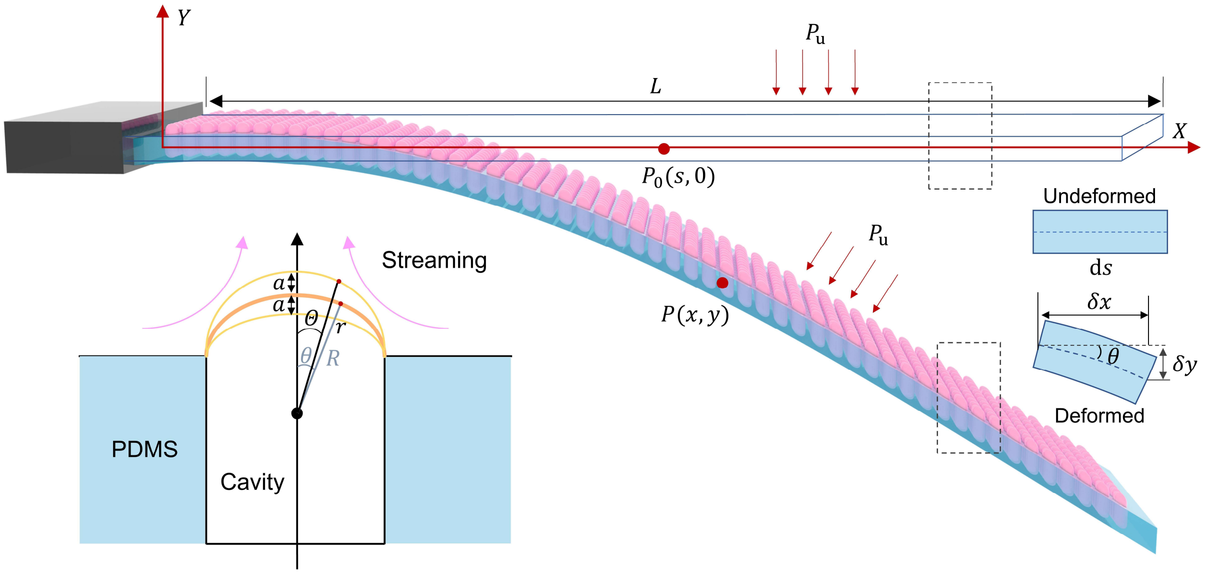

Supplementary Fig. 24 | Modeling of the uniform-size microbubble array artificial muscle. Schematic of a cantilever-like artificial muscle composed of a uniform-size microbubble array, transitioning from the undeformed to the deformed state under microbubble streaming actuation. The inset illustrates the parametrization of an oscillating microbubble. For detailed bubble and muscle parameters, see **Supplementary Notes**.

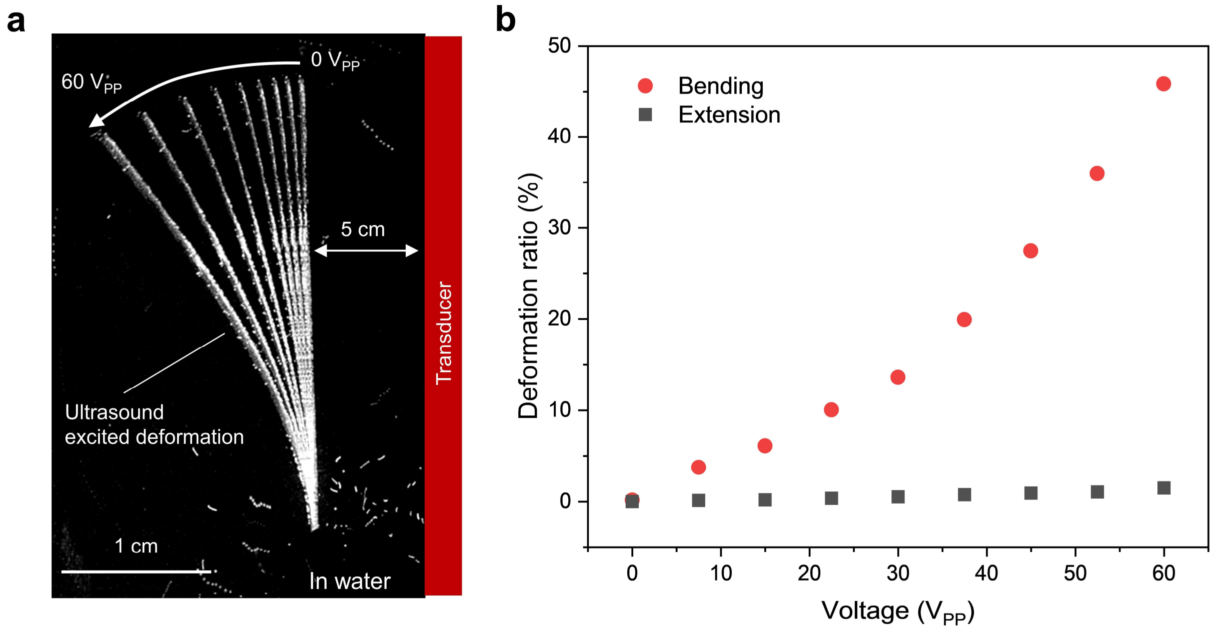

Supplementary Fig. 25 | Deformation of an artificial muscle under ultrasound excitation. a, Superimposed deformation of the uniform-size microbubble array artificial muscle with voltage increased from 0 to 60 V_{PP}, where the excitation frequency was 27.6 kHz. **b**, The bending ratio (red dots) and extension ratio (black dots) along the long axis of the artificial muscle at different excitation voltages, where the measured extension ratio remains below 1.5%.

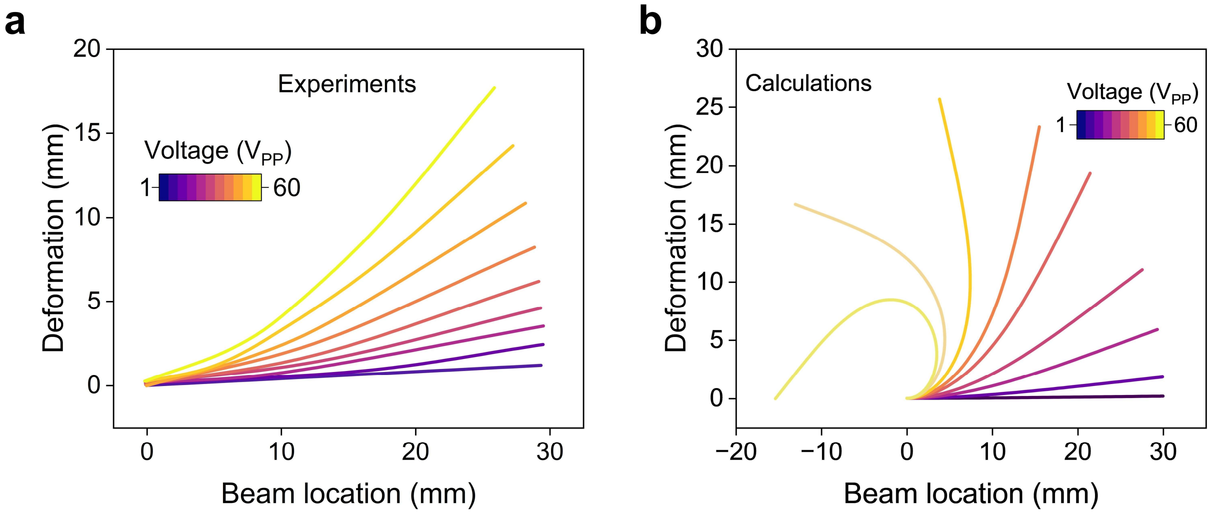

Supplementary Fig. 26 | Comparison of artificial muscle deformation between experimental results and analytical calculations. a, Experimentally observed deformed muscle shapes under voltages ranging from 1 to 60 V_{PP}, extracted from **Supplementary Fig. 6a**. **b**, Corresponding analytical predictions obtained from Eqs. (S9), (S21), and (S22), using force intensities obtained from experimentally measured velocities (**Extended Data Fig. 3b**) under voltages ranging from 1 to 60 V_{PP}. Both experimental and analytical results correspond to a beam measuring approximately 30 mm × 5 mm × 0.33 mm (average thickness), with the Young's modulus of PDMS measured to be 1.6 MPa.

Supplementary Table 1. Comparison data of the response time and gripping ability of grippers for various actuation mechanisms.

Category	Response time (s)	Griping ability (Object/Gripper)	Ref.	Ref. Resources
**Acoustic**	0.1	0.5	Our work	Fig. 3a
**Light**	0.2	0.2	[S5] OM Wani (2017)	Fig. 4d
	60	0.33	[S6] C Ma (2016)	Fig. 4
	35	0.16	[S7] X Zhang (2021)	Fig. 3
**Thermal**	600	1	[S8] W Wei (2018)	Fig. 5
	552	0.833	[S9] AK Mishra (2020)	Fig. 5
	15	0.786	[S10] TG Leong (2009)	Fig. 4
	8	0.63	[S11] Y Roh (2021)	Fig. 3
	1.8	1	[S12] H Shahsavan (2017)	Fig. 4
	1.2	0.2	[S13] S Li (2021)	Fig. 4
	4	0.23	[S14] JC Breger (2015)	Fig. 5
	63	0.8	[S15] C Yao (2015)	Fig. 10
**Magnetic**	0.035	0.8	[S16] X Wang (2020)	Fig. 1
	0.02	0.45	[S17] M Lukas (2024)	Fig. 4
	22	0.3	[S18] H Gu (2023)	Fig. 5
	1	0.28	[S19] Y Jiang (2023)	Fig. 3
**Pneumatic**	0.62	0.37	[S20] Z Zhang (2023)	Fig. 3
	40	0.15	[S21] X Guo (2024)	Fig. 3
	0.065	0.6	[S22] WY Choi (2025)	Fig. 3

Supplementary Table 2. Comparison data of force-to-weight ratios of artificial muscles versus their sizes for various actuation mechanisms.

Category	Size (mm)	Force-to-weight ratio (N/N)	Ref.	Ref. Resources
**Acoustic**	5	35	Our work	Supplementary Fig. S23
**Chemical**	15	2.61	[S23] X Yang (2020)	Movie S7
**Dielectric**	112	1	[S24] SD Gravert (2024)	Fig. 1C
	100	8	[S25] Y Xu (2025)	Fig. 5D
	20	127	[S26] ID Sîrbu (2021)	Fig. S10
**Light**	0.7	0.4	[S27] Y Wang (2022)	Fig. S11
	80	268	[S28] W Ai (2024)	Fig. S4
	33.7	114.9	[S29] H Liu (2022)	Fig. 4F
**Magnetic**	60	1004	[S30] M Seong (2024)	Fig. S11b
	30	64	[S31] Q Ze (2022)	Fig. 3D
**Pneumatic**	100	46.4	[S32] Z Zhang (2019)	Fig. 19
	70	34	[S33] S Zhuo (2020)	Fig. 4
	120	5.9	[S34] SM Mirvakili (2020)	Fig. 6
**Thermal**	10	10	[S35] BE Schubert (2013)	Fig. 1B
	10	450	[S36] Y Yao (2024)	Fig. S25

Supplementary Table 3. Comparison data of relative swimming speeds of swimmers relative to their body length for various actuation mechanisms.

Category	Body length (mm)	Relative speed (BL/s)	Ref.	Ref. Resources
**Acoustic**	50	0.8	Our work	Fig. 4b
	0.07	50	[41] D Ahmed (2015)	Fig. 3
	0.0074	350	[42] L Ren (2019)	Fig. 2D
	0.026	90	[43] A Aghakhani (2020)	Fig. 3D
	0.02	320	[S37] M Nima (2025)	Fig. 2D
	0.03	68	[38] H Han (2024)	Fig. 2N
	0.07	55	[S38] M Nima (2023)	Fig. 4B
	0.18	6.7	[S39] M Kaynak (2017)	Fig. 4
	0.3	0.017	[S40] T Qiu (2014)	Fig. 3a
	0.26	10	[36] C Dillinger (2021)	Fig. 4b
**Biohybrid**	14	1.07	[S41] LK Yong (2017)	Movie S11
	16.3	0.196	[S42] SJ Park (2016)	Fig. 4
	0.27	3	[S43] M Guix (2021)	Abstract
	3.2	0.00017	[S44] O Aydin (2019)	Fig. 4D
	0.0025	0.001	[S45] WC Drennan (2025)	Abstract
**Chemical**	400	0.026	[S46] CA Aubin (2019)	Fig. 5D
	0.003	3.3	[S47] RD Baker (2019)	Fig. 6B
	0.004	2	[S48] S Ketzetzi (2022)	Fig. 2F
	4.3	30	[S49] S Arnaboldi (2021)	Videos 6-8
	0.01	100	[S50] W Gao (2012)	Fig. 3
	0.008	39.75	[S51] L Wang (2016)	Fig. 4
	0.1	3.2	[S52] L Li (2014)	Fig. 5
**Electric**	0.05	0.09	[S53] Y Alapan (2019)	Fig. 3d
	90	1.1	[S54] Z Ye (2025)	Movie S2
	70	0.65	[S55] JD Cortazar (2025)	Abstract
	80	0.11	[S56] H Yang (2025)	Fig. 8G
**Light**	0.0015	14.9	[S57] V Sridhar (2022)	Abstract
	1.23	0.0023	[S58] S Palagi (2016)	Fig. 3c
	0.06	0.0167	[S59] MZ Miskin (2020)	Fig. 4b, c
	10	0.337	[S60] C Yin (2021)	Fig. 3
	5	2.6	[S61] K Hou (2021)	Fig. 3C
	28.2	0.0083	[S62] Y Zhao (2019)	Fig. 5C
**Magnetic**	4	5	[S63] W Hu (2018)	Fig. 2
	6	1.2	[S64] Z Ren (2019)	Fig. 1d
	13	1	[S65] GZ Lum (2016)	Movie S3
	0.009	14.4	[S66] S Tottori (2012)	Video S3
	0.015	4	[S67] X Wang (2018)	Fig. 2
**Pressure**	528	0.36	[S68] DQ Nguyen (2022)	Abstract
	470	0.5	[S69] K Katzschmann (2018)	Fig. 3
	140	3.74	[S70] Y Chi (2022)	Fig. 3

Legends for Supplementary Videos

Video 1. Selective excitation of variable-size microbubble arrays.

Upward microstreaming generated by microbubble arrays with (40 µm, 60 µm, and 80 µm) × 150 µm cavities under ultrasound frequencies of 27.6 kHz, 57.4 kHz, and 76.3 kHz, respectively, at an excitation voltage of 24 V_{PP}. Rectangles indicate the excited regions.

Video 2. Deformations of a uniform-size microbubble array artificial muscle with varying transducer positions.

The transducer was respectively positioned with four distinct orientations: top left panel: directly facing the microbubble-embedded side; top right panel: opposite to it; and perpendicular to the arrays on the left (low left panel) and right (low right panel) sides of the artificial muscle. The side with the microbubble array is marked by a red rectangle. The ultrasound excitation was 80.5 kHz and 60 V_{PP}.

Video 3. Static deformation of a variable-size microbubble array artificial muscle under continuous-frequency ultrasound actuation.

The diameters of the microbubbles in the three different arrays, from top to bottom, are 12 µm, 16 µm, and 66 µm, with corresponding excitation frequencies of 96.5 kHz, 82.3 kHz, and 33.2 kHz. The excitation voltage was 60 V_{PP}.

Video 4. Undulatory deformation of a variable-size microbubble array artificial muscle under sweeping-frequency ultrasound actuation.

The artificial muscle undergoes undulatory motion as the excitation frequency was swept from 20 to 90 kHz, with a sweep time of 1.2 seconds each cycle at 60 V_{PP}.

Video 5. Operation of the soft gripper at arbitrary locations.

The gripper consisting of six to ten uniform-size microbubble array artificial muscles (12 µm × 50 µm cavities), each measuring 10 mm × 0.7 mm × 0.08 mm, was held by a tweezer and functions at arbitrary locations within the acoustic tank at 95.5 kHz and 60 V_{PP}.

Video 6. Soft gripper handling delicate zebrafish larvae.

The gripper was composed of six to ten uniform-size microbubble array artificial muscle tentacles (12 µm × 50 µm cavities), each measuring 10 mm × 0.7 mm × 0.08 mm. When submerged in water, each tentacle contained ~20,000 microbubbles. The ultrasound excitation was 95.5 kHz and 60 V_{PP}. Upon ultrasound stimulation, the tentacles grasped the motile larva; once the acoustic excitation was deactivated, the larva readily swam away.

Video 7. Actuation of an almond.

Rotating of an almond using a conformable robotic skin composed of a uniform-size microbubble array (12 µm × 50 µm cavities), operated at an excitation voltage of 60 V_{PP} and a frequency of 95.5 kHz.

Video 8. Actuation of a piece of grass.

Closing grass blades using a conformable robotic skin composed of a uniform-size microbubble array (12 µm × 50 µm cavities), operated at an excitation voltage of 60 V_{PP} and a frequency of 95.5 kHz.

Video 9. Attachment of the robotic patch to a porcine heart.

The artificial muscle was positioned between an ultrasound transducer (operating at 96 kHz and 60 V_{PP}) and the heart, with a separation of approximately 2.5 cm. It was released from below using tweezers.

Video 10. Multi-modal deformation of the shape transformer.
Multi-modal shape transformation of a circular functional surface as the frequency was swept from 10 to 100 kHz. Each cycle had a sweeping time of 2 seconds, and the input voltage was 60 V_{PP}.

Video 11. Deployment of the encapsulated microbubble-array artificial muscle in a porcine bladder.
Within the porcine bladder, the encapsulated artificial muscle was released and attached to the inner wall.

Video 12. Swimming of the stingraybot.
Fins of the stingraybot were constructed using variable-size microbubble array artificial muscles (12 μm × 50 μm, 16 μm × 50 μm, and 66 μm × 50 μm cavities), arranged vertically from head to tail. The actuation was achieved by sweeping-frequency ultrasound excitation (30–90 kHz, 2-s sweep period, 60 V_{PP}).

Video 13. Undulatory motion and locomotion of the stingraybot in a porcine stomach.
The stingraybot exhibits undulatory motion with a fixed tail and swims untethered.

Video 14. Locomotion of a pre-folded, wheel-like artificial muscle driven by ultrasound inside a porcine stomach and intestine.
The 30 mm × 5 mm × 80 μm muscle, composed of variable-size microbubble arrays (with cavities 12, 16, and 66 μm in diameter, 50 μm in depth), was pre-folded into a wheel-like shape and propels along the stomach surface under sweeping-frequency ultrasound excitation (30–100 kHz, 2-s sweep period, 60 V_{PP}).

Video 15. Acoustic streaming under different ultrasound excitation voltages.
The microbubble array contains 4×4 microbubbles with a diameter of 80 μm. The left and right videos correspond to 10 V_{PP} and 30 V_{PP}, respectively, at a frequency of 27.6 kHz. 6 μm microparticles were used as tracer particles.

Video 16. Response of variable-size microbubble arrays under sweeping-frequency ultrasound excitation.
The actuation was achieved by sweeping the frequency within the range of 10-90 kHz, with a sweep time of 4 seconds each cycle at 30 V_{PP}.

Video 17. Comparison of the deformation of a uniform-size microbubble array artificial muscle without and with microbubbles.
Without microbubbles, the excitation frequency was in the range of 1 – 100 kHz, with a sweeping time of 3 seconds per cycle at 60 V_{PP}. With 40 μm microbubbles, the excitation frequency and voltage were 9.5 kHz and 60 V_{PP}.

Video 18. Comparison between the stingraybot fin deformations with/without microbubbles.
The left panel shows the fin without embedded microbubbles, while the right panel includes the microbubble arrays. Actuation was achieved using a sweeping-frequency ultrasound excitation (60–100 kHz, 1.5-s sweep period, 60 V_{PP}). In both experiments, the stingraybot was fixed to the bottom of the acoustic tank.

Supplementary References

S1. Gelderblom, H. *et al.* Oscillations of a gas pocket on a liquid-covered solid surface. *Phys. Fluids* **24**, 122105 (2012).
S2. Gritsenko, D. *et al.* Vibrational modes prediction for water-air bubbles trapped in circular microcavities. *Phys. Fluids* **30**, 082005 (2018).
S3. Schnitzer, O., Brandão, R. & Yariv, E. Acoustics of bubbles trapped in microgrooves: from isolated subwavelength resonators to superhydrophobic metasurfaces. *Phys. Rev. B* **99**, 195155 (2019).
S4. Dolev, A., Kaynak, M. & Sakar, M. S. Dynamics of entrapped microbubbles with multiple openings. *Phys. Fluids* **34**, 012110 (2022).
S5. Wani, O., Zeng, H. & Priimagi, A. A light-driven artificial flytrap. *Nat. Commun.* **8**, 15546 (2017).
S6. Ma, C. *et al.* A multiresponsive anisotropic hydrogel with macroscopic 3D complex deformations. *Adv. Funct. Mater.* **26**, 8670–8676 (2016).
S7. Zhang, X. *et al.* Optically- and thermally-responsive programmable materials based on carbon nanotube-hydrogel polymer composites. *Nano Lett.* **11**, 3239–3244 (2011).
S8. Wang, W. *et al.* Soft tendril-inspired grippers: shape morphing of programmable polymer–paper bilayer composites. *ACS Appl. Mater. Interfaces* **10**, 10419–10427 (2018).
S9. Mishra, A. K. *et al.* Autonomic perspiration in 3D-printed hydrogel actuators. *Sci. Robot.* **5**, eaaz3918 (2020).
S10. Leong, T. G. *et al.* Tetherless thermobiochemically actuated microgrippers. *Proc. Natl Acad. Sci. USA* **106**, 703–708 (2009).
S11. Roh, Y. *et al.* Vital signal sensing and manipulation of a microscale organ with a multifunctional soft gripper. *Sci. Robot.* **6**, eabi6774 (2021).
S12. Shahsavan, H. *et al.* Thermally active liquid crystal network gripper mimicking the self-peeling of gecko toe pads. *Adv. Mater.* **29**, 1604021 (2017).
S13. Li, S. *et al.* Digital light processing of liquid crystal elastomers for self-sensing artificial muscles. *Sci. Adv.* **7**, eabg3677 (2021).
S14. Breger, J. C. *et al.* Self-folding thermo-magnetically responsive soft microgrippers. *ACS Appl. Mater. Interfaces* **7**, 3398–3405 (2015).
S15. Yao, C. *et al.* Poly(N-isopropylacrylamide)–clay nanocomposite hydrogels with responsive bending property as temperature-controlled manipulators. *Adv. Funct. Mater.* **25**, 2980–2991 (2015).
S16. Wang, X. *et al.* Untethered and ultrafast soft-bodied robots. *Commun. Mater.* **1**, 67 (2020).
S17. Masjosthusmann, L. *et al.* Miniaturized variable stiffness gripper locally actuated by magnetic fields. *Adv. Intell. Syst.* **6**, 2400037 (2024).
S18. Gu, H. *et al.* Self-folding soft-robotic chains with reconfigurable shapes and functionalities. *Nat. Commun.* **14**, 1263 (2023).
S19. Yan, J. *et al.* Ultracompact single-nanowire-morphed grippers driven by vectorial Lorentz forces for dexterous robotic manipulations. *Nat. Commun.* **14**, 3786 (2023).
S20. Zhang, Z. *et al.*, Soft and lightweight fabric enables powerful and high-range pneumatic actuation. *Sci. Adv.* **9**, eadg1203 (2023).
S21. Guo, X. *et al.*, Powerful UAV manipulation via bioinspired self-adaptive soft self-contained gripper. *Sci. Adv.* **10**, eadn6642 (2024).
S22. Choi, W. Y. *et al.* A hyperelastic torque-reversal mechanism for soft joints with compression-responsive transient bistability. *Sci. Robot.* **10**, eado7696 (2025).
S23. Yang, X. *et al.*, An 88-milligram insect-scale autonomous crawling robot driven by a catalytic artificial muscle. *Sci. Robot*. **5**, eaba0015 (2020).
S24. Gravert, S.-D. *et al.* Low-voltage electrohydraulic actuators for untethered robotics. *Sci. Adv.* **10**, eadi9319 (2024).
S25. Xu, Y. *et al.* Monolithic electrostatic actuators with independent stiffness modulation. *Nat. Commun.* **16**, 1174 (2025).
S26. Sîrbu, I.-D. *et al.* Electrostatic bellow muscle actuators and energy harvesters that stack up. *Sci.*

Robot. **6**, eaaz5796 (2021).
S27. Wang, Y. *et al.* In situ integrated microrobots driven by artificial muscles built from biomolecular motors. *Sci. Robot.* **7**, eaba8212 (2022).
S28. Ai, W. *et al.* Miniaturized and untethered McKibben muscles based on photothermal-induced gas–liquid transformation. *Nat. Commun.* **15**, 1329 (2024).
S29. Liu, H. *et al.* Shape-programmable, deformation-locking, and self-sensing artificial muscle based on liquid crystal elastomer and low-melting-point alloy. *Sci. Adv.* **8**, eabn5722 (2022).
S30. Seong, M. *et al.* Multifunctional magnetic muscles for soft robotics. *Nat. Commun.* **15**, 7929 (2024).
S31. Ze, Q. *et al.* Spinning-enabled wireless amphibious origami millirobot. *Nat. Commun.* **13**, 3118 (2022).
S32. Zhang, Z. *et al.* Design and modeling of a parallel-pipe-crawling pneumatic soft robot. *IEEE Access* **7**, 134301–134317 (2019).
S33. Zhuo, S. *et al.* Complex multiphase organohydrogels with programmable mechanics toward adaptive soft-matter machines. *Sci. Adv.* **6**, eaax1464 (2020).
S34. Mirvakili, S. M. *et al.* Actuation of untethered pneumatic artificial muscles and soft robots using magnetically induced liquid-to-gas phase transitions. *Sci. Robot.* **5**, eaaz4239 (2020).
S35. Schubert, B. E. & Floreano, D. Variable stiffness material based on rigid low-melting-point-alloy microstructures embedded in soft PDMS. *RSC Adv.* **3**, 24671–24679 (2013).
S36. Yao, Y. *et al.* Programming liquid crystal elastomers for multistep ambidirectional deformability. *Science* **386**, 1161–1168 (2024).
S37. Mahkam, N. *et al.* Multiorifice acoustic microrobot for boundary-free multimodal 3D swimming. *Proc. Natl Acad. Sci. USA* **122**, e2417111122 (2025).
S38. Mahkam, N. *et al.* Acoustic streaming-induced multimodal locomotion of bubble-based microrobots. *Adv. Sci.* **10**, 2304233 (2023).
S39. Kaynak, M. *et al.* Acoustic actuation of bioinspired microswimmers. *Lab Chip* **17**, 395–400 (2017).
S40. Qiu, T. *et al.* Swimming by reciprocal motion at low Reynolds number. *Nat. Commun.* **5**, 5119 (2014).
S41. Lee, K. Y. *et al.* An autonomously swimming biohybrid fish designed with human cardiac biophysics. *Science* **375**, 639–647 (2022).
S42. Park, S.-J. *et al.* Phototactic guidance of a tissue-engineered soft-robotic ray. *Science* **353**, 158–162 (2016).
S43. Guix, M. *et al.* Biohybrid soft robots with self-stimulating skeletons. *Sci. Robot.* **6**, eabe7577 (2021).
S44. Aydin, O. *et al.* Neuromuscular actuation of biohybrid motile bots. *Proc. Natl Acad. Sci. USA* **116**, 19841–19847 (2019).
S45. Drennan, W. C. *et al.*, A forward-engineered, muscle-driven soft robotic swimmer. *Sci. Adv.* **11**, eadu8634 (2025).
S46. Aubin, C. A. *et al.* Electrolytic vascular systems for energy-dense robots. *Nature* **571**, 51–57 (2019).
S47. Baker, R. D. *et al.* Shape-programmed 3D printed swimming microtori for the transport of passive and active agents. *Nat. Commun.* **10**, 4932 (2019).
S48. Ketzetzi, S. *et al.* Activity-induced interactions and cooperation of artificial microswimmers in one-dimensional environments. *Nat. Commun.* **13**, 1772 (2022).
S49. Arnaboldi, S. *et al.* Direct dynamic read-out of molecular chirality with autonomous enzyme-driven swimmers. *Nat. Chem.* **13**, 1241–1247 (2021).
S50. Gao, W., Uygun, A. & Wang, J. Hydrogen-bubble-propelled zinc-based microrockets in strongly acidic media. *J. Am. Chem. Soc.* **134**, 897–900 (2012).
S51. Wang, L. *et al.* Self-propelled manganese oxide-based catalytic micromotors for drug delivery. *RSC Adv.* **6**, 65624–65630 (2016).

S52. Li, L. *et al.* Hydrodynamics and propulsion mechanism of self-propelled catalytic micromotors: model and experiment. *Soft Matter* **10**, 7511–7518 (2014).
S53. Alapan, Y. *et al.* Shape-encoded dynamic assembly of mobile micromachines. *Nat. Mater.* **18**, 1244–1251 (2019).
S54. Ye, Z. *et al.* Ocyropsis-inspired fast-swimming transparent soft robots. *Adv. Funct. Mater.* **34**, 2421522 (2025)
S55. Cortazar, J. D. & Lazarus, N. Creating a softer RoboSquid: liquid-metal-based compliant pumps for pulsed jet propulsion. *Adv. Mater. Technol.* **10**, 2401372 (2025).
S56. Yang, H. *et al.* Weaving liquid crystal elastomer fiber actuators for multifunctional soft robotics. *Sci. Adv.* **11**, eads3058 (2025).
S57. Sridhar, V. *et al.* Light-driven carbon nitride microswimmers with propulsion in biological and ionic media and responsive on-demand drug delivery. *Sci. Robot.* **7**, eabm1421 (2022).
S58. Palagi, S. *et al.* Structured light enables biomimetic swimming and versatile locomotion of photoresponsive soft microrobots. *Nat. Mater.* **15**, 647–653 (2016).
S59. Miskin, M. Z. *et al.* Electronically integrated, mass-manufactured, microscopic robots. *Nature* **584**, 557–561 (2020).
S60. Yin, C. *et al.* Visible light-driven jellyfish-like miniature swimming soft robot. *ACS Appl. Mater. Interfaces* **13**, 47147–47154 (2021).
S61. Hou, K. *et al.* Programmable light-driven swimming actuators via wavelength signal switching. *Sci. Adv.* **7**, eabh3051 (2021).
S62. Zhao, Y. *et al.* Soft phototactic swimmer based on self-sustained hydrogel oscillator. *Sci. Robot.* **4**, eaax7112 (2019).
S63. Hu, W. *et al.* Small-scale soft-bodied robot with multimodal locomotion. *Nature* **554**, 81–85 (2018).
S64. Ren, Z. *et al.* Multi-functional soft-bodied jellyfish-like swimming. *Nat. Commun.* **10**, 2703 (2019).
S65. Lum, G. Z. *et al.* Shape-programmable magnetic soft matter. *Proc. Natl Acad. Sci. USA* **113**, E6007–E6015 (2016).
S66. Tottori, S. *et al.* Magnetic helical micromachines: fabrication, controlled swimming, and cargo transport. *Adv. Mater.* **24**, 811–816 (2012).
S67. Wang, X. *et al.* Surface-chemistry-mediated control of individual magnetic helical microswimmers in a swarm. *ACS Nano* **12**, 6210–6217 (2018).
S68. Nguyen, D. Q. & Ho, V. A. Anguilliform swimming performance of an eel-inspired soft robot. *Soft Robot.* **9**, 425–439 (2022).
S69. Katzschmann, R. K. *et al.* Exploration of underwater life with an acoustically controlled soft robotic fish. *Sci. Robot.* **3**, eaar3449 (2018).
S70. Chi, Y. *et al.* Snapping for high-speed and high-efficient butterfly stroke–like soft swimmer. *Sci. Adv.* **8**, eadd3788 (2022).
