# Supplementary material for: Ultrasound-driven programmable artificial muscles
Source: Nature. 2025 Oct 29;646(8087):1096–104. doi: 10.1038/s41586-025-09650-3 (PMC12571877; doi:10.1038/s41586-025-09650-3)
Supplement: Supplementary file 2 — Reporting Summary [file 41586_2025_9650_MOESM2_ESM.pdf]

nature portfolio

Corresponding author(s): Daniel Ahmed

Last updated by author(s): Aug 28, 2025

Reporting Summary

Nature Portfolio wishes to improve the reproducibility of the work that we publish. This form provides structure for consistency and transparency in reporting. For further information on Nature Portfolio policies, see our Editorial Policies and the Editorial Policy Checklist.

Statistics

For all statistical analyses, confirm that the following items are present in the figure legend, table legend, main text, or Methods section.

n/a	Confirmed	
☐	☒	The exact sample size (*n*) for each experimental group/condition, given as a discrete number and unit of measurement
☐	☒	A statement on whether measurements were taken from distinct samples or whether the same sample was measured repeatedly
☒	☐	The statistical test(s) used AND whether they are one- or two-sided<br>*Only common tests should be described solely by name; describe more complex techniques in the Methods section.*
☒	☐	A description of all covariates tested
☒	☐	A description of any assumptions or corrections, such as tests of normality and adjustment for multiple comparisons
☒	☐	A full description of the statistical parameters including central tendency (e.g. means) or other basic estimates (e.g. regression coefficient) AND variation (e.g. standard deviation) or associated estimates of uncertainty (e.g. confidence intervals)
☒	☐	For null hypothesis testing, the test statistic (e.g. *F*, *t*, *r*) with confidence intervals, effect sizes, degrees of freedom and *P* value noted<br>*Give P values as exact values whenever suitable.*
☒	☐	For Bayesian analysis, information on the choice of priors and Markov chain Monte Carlo settings
☒	☐	For hierarchical and complex designs, identification of the appropriate level for tests and full reporting of outcomes
☒	☐	Estimates of effect sizes (e.g. Cohen's *d*, Pearson's *r*), indicating how they were calculated

Our web collection on statistics for biologists contains articles on many of the points above.

Software and code

Policy information about availability of computer code

Data collection	A high-speed camera (Chronos 1.4, Kron Technologies) mounted on a Zeiss Axiovert 200M microscope, a Blackmagic camera (Pocket Cinema Camera 4K), a Canon camera (EOS 6D) and an endoscope (Fuan) were used to acquire images and videos.
Data analysis	All the image and video processing was performed using ImageJ 1.53k (v-Java 1.8.0_172 (64-bit)). Simulation and statistical analysis were conducted using Comsol Multiphysics (v6.1, Burlington, MA), MATLAB (version R2021b), Originlab (version Origin 2023) and Excel (version 16.54).

For manuscripts utilizing custom algorithms or software that are central to the research but not yet described in published literature, software must be made available to editors and reviewers. We strongly encourage code deposition in a community repository (e.g. GitHub). See the Nature Portfolio guidelines for submitting code & software for further information.

Data

Policy information about availability of data

All manuscripts must include a data availability statement. This statement should provide the following information, where applicable:

- Accession codes, unique identifiers, or web links for publicly available datasets
- A description of any restrictions on data availability
- For clinical datasets or third party data, please ensure that the statement adheres to our policy

The datasets that support the findings of this study are available within the paper. Correspondence and requests for materials should be addressed to Daniel Ahmed. The time frame for response is 12 months.

Research involving human participants, their data, or biological material

Policy information about studies with human participants or human data. See also policy information about sex, gender (identity/presentation), and sexual orientation and race, ethnicity and racism.

Reporting on sex and gender	N/A
Reporting on race, ethnicity, or other socially relevant groupings	N/A
Population characteristics	N/A
Recruitment	N/A
Ethics oversight	N/A

Note that full information on the approval of the study protocol must also be provided in the manuscript.

Field-specific reporting

Please select the one below that is the best fit for your research. If you are not sure, read the appropriate sections before making your selection.

☒ Life sciences ☐ Behavioural & social sciences ☐ Ecological, evolutionary & environmental sciences

For a reference copy of the document with all sections, see nature.com/documents/nr-reporting-summary-flat.pdf

Life sciences study design

All studies must disclose on these points even when the disclosure is negative.

Sample size	No formal statistical method was used to predetermine sample size. For zebrafish experiments, the number of larvae (Danio rerio, WIK strain, 2–5 days post fertilization) was chosen to ensure reproducibility and consistency with prior studies. For porcine experiments, ex vivo tissues were obtained from animals slaughtered for food production; sample sizes were determined by tissue availability. In both cases, the chosen sample sizes were sufficient to yield reproducible results across independent experiments.
Data exclusions	No data acquired for quantitative analysis were excluded.
Replication	All replication attempts were successful under the reported conditions. Gripping experiments with zebrafish larvae consistently succeeded across individuals within the defined gripping range. Similarly, porcine ex vivo tissue experiments were successfully replicated using samples obtained from different animals. A minimum of five times of experiments have been conducted independently for a vast majority of conditions.
Randomization	For zebrafish experiments, larvae (Danio rerio, WIK strain, 2–5 days post fertilization) were allocated to experiments based on developmental stage and availability within the defined age window. No formal randomization was applied, as larvae within this stage are developmentally homogeneous. For porcine ex vivo experiments, tissues were obtained from animals slaughtered for food production and used according to availability. Allocation into groups was not relevant, as the experiments did not involve comparison between treatment groups. In both cases, experimental conditions were standardized to control for potential covariates.
Blinding	Blinding was not relevant, as experiments did not involve allocation into treatment groups; all zebrafish larvae and porcine ex vivo tissues were handled under the same experimental conditions, respectively.

Reporting for specific materials, systems and methods

We require information from authors about some types of materials, experimental systems and methods used in many studies. Here, indicate whether each material, system or method listed is relevant to your study. If you are not sure if a list item applies to your research, read the appropriate section before selecting a response.

Materials & experimental systems

n/a	Involved in the study
☒	☐ Antibodies
☒	☐ Eukaryotic cell lines
☒	☐ Palaeontology and archaeology
☐	☒ Animals and other organisms
☒	☐ Clinical data
☒	☐ Dual use research of concern
☐	☒ Plants

Methods

n/a	Involved in the study
☒	☐ ChIP-seq
☒	☐ Flow cytometry
☒	☐ MRI-based neuroimaging

Animals and other research organisms

Policy information about studies involving animals; ARRIVE guidelines recommended for reporting animal research, and Sex and Gender in Research

Laboratory animals	Zebrafish larvae (Danio rerio) of the WIK strain, 2-5 days post fertilization.
Wild animals	No wild animals were used in this study.
Reporting on sex	Larvae were used before sex determination takes place. Hence all larvae are neither male or female, but still sex in-determinant. Ex vivo tissues were obtained from porcine slaughtered for food production. Both male and female animals were used; sex was not specifically reported.
Field-collected samples	The study did not include field-collected samples.
Ethics oversight	Experiments on larvae until 5 dpf do not fall under animal welfare regulations. Husbandry and housing was approved by local authorities. (Kantonales Veterinäramt TV4206). Porcine hearts, stomach, intestine, ribs and blood were obtained from a licensed abattoir. As the study involved only ex vivo tissues from animals slaughtered for food production, no ethical approval was required.

Note that full information on the approval of the study protocol must also be provided in the manuscript.

Dual use research of concern

Policy information about dual use research of concern

Hazards

Could the accidental, deliberate or reckless misuse of agents or technologies generated in the work, or the application of information presented in the manuscript, pose a threat to:

No	Yes
☒	☐ Public health
☒	☐ National security
☒	☐ Crops and/or livestock
☒	☐ Ecosystems
☒	☐ Any other significant area

Experiments of concern

Does the work involve any of these experiments of concern:

No	Yes	
☒	☐	Demonstrate how to render a vaccine ineffective
☒	☐	Confer resistance to therapeutically useful antibiotics or antiviral agents
☒	☐	Enhance the virulence of a pathogen or render a nonpathogen virulent
☒	☐	Increase transmissibility of a pathogen
☒	☐	Alter the host range of a pathogen
☒	☐	Enable evasion of diagnostic/detection modalities
☒	☐	Enable the weaponization of a biological agent or toxin
☒	☐	Any other potentially harmful combination of experiments and agents

Plants

Seed stocks	Commercially available tea leaves and almonds, intended for human consumption, were used in the experiments. As the material was edible and non-living, no ethical approval was required.
Novel plant genotypes	This study did not include novel plant genotypes.
Authentication	This study did not require authentication procedures.
